# Quantitative Comparison of the Light-to-Heat Conversion Efficiency in Nanomaterials Suitable for Photothermal Therapy

**DOI:** 10.1021/acsami.2c08013

**Published:** 2022-07-18

**Authors:** Agnieszka Paściak, Riccardo Marin, Lise Abiven, Aleksandra Pilch-Wróbel, Małgorzata Misiak, Wujun Xu, Katarzyna Prorok, Oleksii Bezkrovnyi, Łukasz Marciniak, Corinne Chanéac, Florence Gazeau, Rana Bazzi, Stéphane Roux, Bruno Viana, Vesa-Pekka Lehto, Daniel Jaque, Artur Bednarkiewicz

**Affiliations:** † Institute of Low Temperature and Structure Research, Polish Academy of Sciences, Okólna 2, 50-422 Wroclaw, Poland; ‡ Nanomaterials for Bioimaging Group (nanoBIG), Departamento de Física de Materiales, Facultad de Ciencias, Universidad Autónoma de Madrid, C/Francisco Tomás y Valiente 7, Madrid 28049, Spain; § Sorbonne Université, CNRS, Laboratoire de Chimie de la Matière Condensée de Paris, UMR 7574, 4 Place Jussieu, F-75005 Paris, France; ∥ Department of Applied Physics, University of Eastern Finland, 70211 Kuopio, Finland; ⊥ Université Paris Cité, CNRS, Matière et Systèmes Complexes, F75006 Paris, France; # Institut UTINAM, UMR 6213 CNRS-UBFC, Université Bourgogne Franche-Comté, 16 route de Gray, 25030 Besançon, Cedex, France; ∇ Chimie ParisTech, CNRS, Institut de Recherche de Chimie Paris, PSL Research University, 11 rue P. et M. Curie, F-75231 Paris, Cedex 05, France

**Keywords:** photothermal conversion efficiency, nanoheaters, photothermal treatment, gold nanoparticles, lanthanide-doped nanomaterials, porous silicon, semiconductor nanocrystals

## Abstract

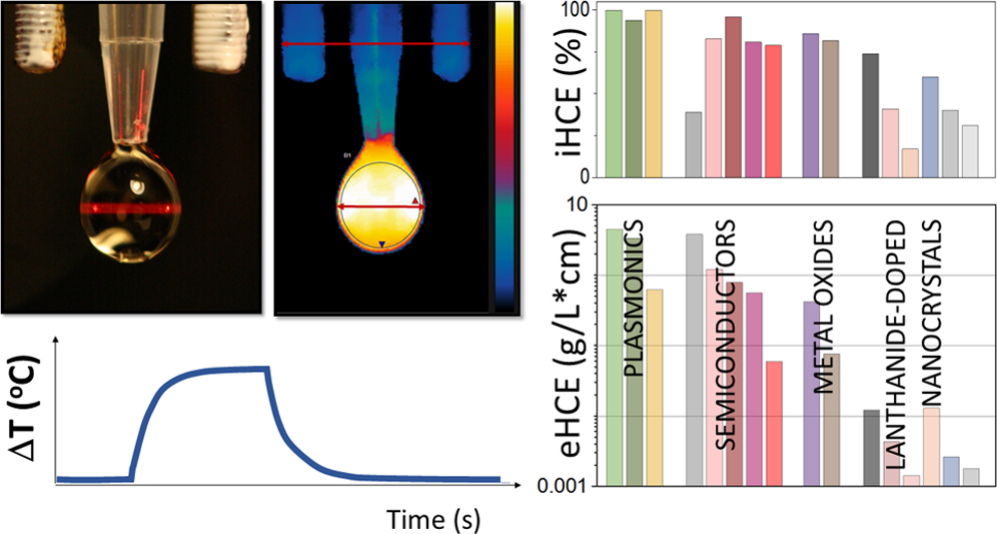

Functional colloidal nanoparticles capable of converting between various energy types are finding an increasing number of applications. One of the relevant examples concerns light-to-heat-converting colloidal nanoparticles that may be useful for localized photothermal therapy of cancers. Unfortunately, quantitative comparison and ranking of nanoheaters are not straightforward as materials of different compositions and structures have different photophysical and chemical properties and may interact differently with the biological environment. In terms of photophysical properties, the most relevant information to rank these nanoheaters is the light-to-heat conversion efficiency, which, along with information on the absorption capacity of the material, can be used to directly compare materials. In this work, we evaluate the light-to-heat conversion properties of 17 different nanoheaters belonging to different groups (plasmonic, semiconductor, lanthanide-doped nanocrystals, carbon nanocrystals, and metal oxides). We conclude that the light-to-heat conversion efficiency alone is not meaningful enough as many materials have similar conversion efficiencies—in the range of 80–99%—while they significantly differ in their extinction coefficient. We therefore constructed their qualitative ranking based on the external conversion efficiency, which takes into account the conventionally defined light-to-heat conversion efficiency and its absorption capacity. This ranking demonstrated the differences between the samples more meaningfully. Among the studied systems, the top-ranking materials were black porous silicon and CuS nanocrystals. These results allow us to select the most favorable materials for photo-based theranostics and set a new standard in the characterization of nanoheaters.

## 1 Introduction

Photothermal conversion nanoparticles, referred to as nanoheaters (NHs), allow one to increase the temperature of their surroundings in a spatially localized and contactless manner, which opens new opportunities in many areas. For example, optical NHs can be applied in technology areas such as solar light energy harvesting, photocatalysts or photoactuators,^1^ as well as in biotechnology in the treatment of dentin hypersensitivity^2^ or antibacterial therapy.^3,4^ Additionally, NHs have shown great prospects in cancer photothermal therapy (PTT),^5^ especially after the first report of the successful application of NHs in clinical trials for the treatment of prostate cancer.^6^ Depending on the temperature range, the PTT takes advantage of the fact that, unlike healthy cells, cancer cells are specifically more sensitive to overheating beyond 41 °C (hyperthermia range), or the affected cells are damaged by thermal ablation (temperatures above 48 °C).^7^

Despite the great interest and significant progress in the field of optically stimulated heating nanomaterials, there are numerous factors, materials, and methods to be optimized to enable practical applications of PTT in clinics. First of all, as for any nanomaterial-based therapeutic and diagnostic approach, the NH’s cytotoxicity must be verified before it could be considered for clinical trials.^8−10^ Also, NHs should accumulate and reside in targeted tissues long enough to enable conducting efficient therapy. Moreover, the suitable NHs for PTT should ideally be biodegradable or excretable, which would ensure better biosafety.^11^ Moreover, the safety of stimulus must be assured—in particular, a light dose and appropriate wavelength must ensure safe and deep treatment. For example, to overcome the absorption of water and tissue components, the most appropriate approach is to select NHs whose absorption lies in one of the biological spectral windows (e.g., NIR-I: 700–980 nm, NIR-II: 1000–1400 nm).^7^ Among them, the first biological window, NIR-I, is preferable due to the significantly lower absorption of water at the PTT photoexcitation wavelengths. From the photophysics and materials science perspective, NHs should exhibit a high absorption coefficient at the irradiation wavelength and high light-to-heat conversion efficiency (i.e., internal light-to-heat conversion efficiency; iHCE), which is defined as the capability to convert the absorbed energy specifically into heat. Moreover, their size should not exceed 200 nm to avoid undesired effects, which might appear after injection, such as thrombus, occlusions, or kidney blocks.^12^ Knowledge of these physical parameters allows for an initial assessment of the suitability of the material as a PTT NH before conducting critical experiments on animals and clinical trials.

Different classes of NHs have been already proposed, including organic (e.g., dyes,^13−15^ polymers^16,17^) and inorganic systems. In the latter class, typical materials are plasmonic nanoparticles (e.g., AuNPs,^18−20^ Ag NPs,^21^ Cu_2–*x*_S^22^), semiconductors (e.g., quantum dots,^23^ silicon-based nanoparticles,^24^ or titanium-based nanoparticles^25^), and lanthanide-doped nanoparticles,^26,27^ along with carbon^13,28,29^ and metal oxide^11,30^ NHs. Given the differences in their physicochemical properties, there is an urgent need for a comparison of various nanomaterials in view of their different morphologies, sizes, surface chemistries as well as physical and chemical compositions or properties such as iHCE.

iHCE conventionally determines how much of the absorbed ultraviolet, visible, or near-infrared (UV/vis/NIR) radiation will be converted into heat. Typically, Roper’s model is used for that purpose, which is based on the spontaneous cooling profile.^31^ However, in our previous work,^32^ we proved that the analysis of the heating profile (Wang’s model^20^) leads to more consistent results for various measurement configurations. In Wang’s model, in the case of negligible heating of the solvent, the iHCE is determined from the equation
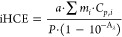
1where ∑*m*_*i*_·*C*_*p*,*i*_ is a sum of the product of the effective mass and heat capacity of sample and experimental system components, *P*[*W*] is the power of the laser beam illuminating the sample, and *A*_λ_ is the absorbance of the sample. The parameter *a*[*K*/*s*] describes how the temperature of a sample changes per unit time under the influence of absorbed energy, and the parameter *b*[1/*s*] is the rate constant. These parameters are determined by fitting the growing part of the heating–cooling kinetic profiles with the equation
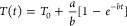
2Because iHCE is often determined with arbitrary and not justified assumptions (e.g., considering the mass of a sample holder instead of the effective mass of the NH), which leads to disparate results for the same material,^32^ it is necessary to be able to qualitatively compare the available materials using a standardized method. We demonstrated that the droplet-based measuring system not only requires a small amount of sample but also offers time-efficient measurement unlike most other conventional systems. Using the setup and methods developed previously,^32^ in this work, we performed a systematic and quantitative comparison of colloidal, light-to-heat-converting NHs belonging to different classes aiming to rank them according to their iHCE. It must be clearly stated that these various NHs convert the delivered photoexcitation energy to heat, exploiting various physical mechanisms (discussed in Section 3). In particular, we have examined various classes of NH colloidal nanoparticles:

(i) Plasmonic: gold nanorods (AuNRs) and copper sulfide coated by glutathione (CuS@GSH) and by citrate (CuS@cit);
(ii) Lanthanide-doped nanoparticles: Nd, Nd/Sm, and Nd/Dy codoped NaYF_4_ nanoparticles;
(iii) Semiconductor: silver sulfide NHs covered by polyethylene glycol (Ag_2_S@PEG), dithiolated diethylenetriamine pentaacetic acid (Ag_2_S@DTDTPA), mercaptoundecanoic acid (Ag_2_S@MUA), Ag-Ag_2_S dimers, and black porous silicon (BPSi);
(iv) Carbon: carbon dots (CDs); and
(v) Metal oxide: maghemite γ-Fe_2_O_3_ and maghemite nanoflowers decorated with gold nanoparticles Au: γ-Fe_2_O_3_-Au.

In the course of the study, we realized that the iHCE, which is an internal heating efficiency, is not sufficient to rank the NH materials for practical applications in PTT. Therefore, an external heating efficiency (eHCE) figure of merit was proposed, similar to the brightness in photoluminescence metrology. Moreover, to understand which factors can affect the efficiency of light-to-heat conversion for selected NHs, we examined different coatings and codoping in the case of rare earth ions. For materials with broad absorption bands, we have systematically determined how the iHCE and eHCE depend on wavelength, which may enhance the understanding of their physical properties and will also allow for optimal wavelength selection for therapy purposes.

## 2 Materials and Methods

### 2.1 Synthesis Procedures

Since an extensive range of materials is presented in this paper, descriptions of the synthesis and a list of the reactants used are included in the Supporting Information (Descriptions S1 and S2).

### 2.2 Material Characterization

The morphology of the samples, AuNRs, NaNdF_4_:Dy@PAA, and carbon dots, on the one hand, and Ag_2_S, γ-Fe_2_O_3_, and of γ-Fe_2_O_3_-Au, on the other hand, was determined by transmission electron microscopy (TEM), using a Philips CM-20 Super-Twin instrument operating at 160 kV and an FEI Tecnai Spirit G2 instrument at an acceleration voltage of 120.0 kV, respectively. Before the measurement, samples were diluted with a suitable solvent and dispersed in an ultrasonic bath; then, a droplet of the suspension was deposited on a copper grid coated with a carbon film. BPSi was imaged with high-resolution transmission electron microscopy (HR-TEM) (JEOL JEM2100F). The morphologies of samples CuS and Ag-Ag_2_S were investigated using a transmission electron microscope (TEM, JEOL JEM1400 Flash) operating at 100 kV. For TEM observations, the particles were precipitated with isopropanol (iPrOH), recovered by means of centrifugation (30,000*g* for 20 min at 4 °C), and washed once with a mixture of water and iPrOH, before being redispersed in water.

Powder diffraction data of AuNRs, NaNdF_4_:Dy@PAA, and C-dots were collected on an X’Pert PRO X-ray diffractometer equipped with a PIXcel ultrafast line detector, a focusing mirror, and soller slits for Cu Kα radiation. The XRD profiles of BPSi and γ-Fe_2_O_3_ were measured using Cu Kα radiation on a Bruker D8 Advance; in the case of BPSi, measurements were performed with a zero-background sample holder. For Ag_2_S diffractometer Bruker D8 discover equipped with a EIGER2 R 500K 2D detector was used. For CuS and Ag-Ag_2_S nanoparticles, X-ray powder diffraction (XRPD) measurements were performed on a Rigaku D/max-γB diffractometer working in the Bragg–Brentano geometry (θ–2θ) with a step of 0.03° in the 20–60° range. A filtered Cu Kα radiation (λ = 1.5418 Å) was used.

Absorption spectra were obtained in the transmission mode using a Cary Varian 5E UV–vis–NIR spectrometer. In the UV region and the vis/NIR region, a deuterium and a halogen lamp were, respectively, used as excitation sources. In the UV and visible ranges, the R928 photomultiplier was used as a detector, and a cooled PbS detector was used for the NIR region. For CuS and Ag-Ag_2_S nanoparticles, optical extinction spectra were recorded at room temperature with a UV–vis–NIR spectrophotometer (Perkin Elmer Lambda1050) using a 3 nm step.

Sample concentration was estimated by synthesis conditions when it was possible (in the case of Ln^3+^-doped materials, CDs, CuS, γ-Fe_2_O_3_-Au), by redox titration of Fe^3+^ using Cr_2_O_7_^2–^ for γ-Fe_2_O_3_, or by evaporating and weighing the material (AuNRs, BPSi, Ag_2_S, Ag-Ag_2_S).

#### 2.2.1 Light-to-Heat Conversion Efficiency Measurements: Procedure

**Figure 1:**
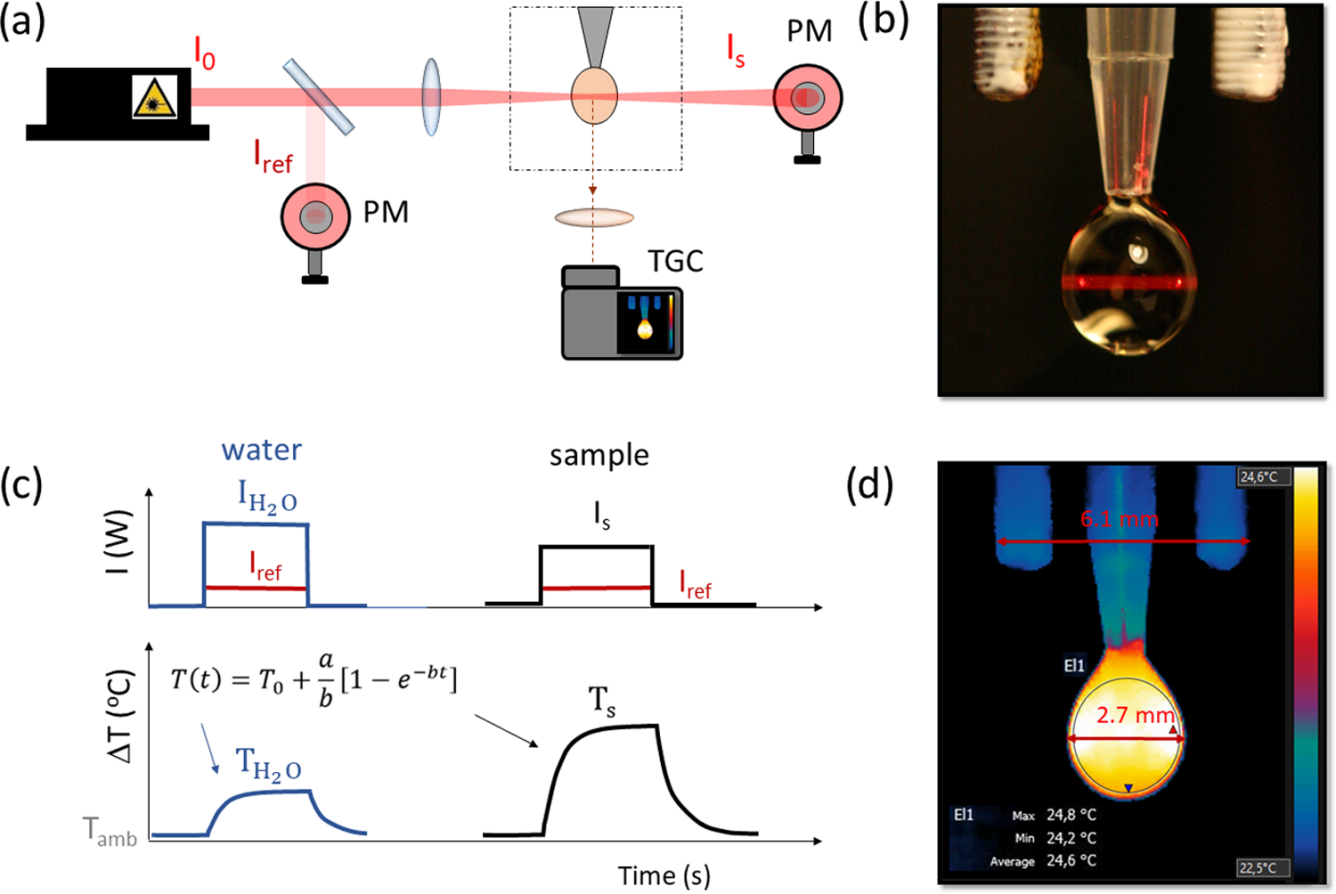
Schematic setup and methodology for measuring the efficiency of light-to-heat conversion of colloidal nanomaterials. (a) Experimental “droplet” setup (PM, power meter; TGC, thermographic camera). (b) Photography of a typical droplet irradiated by a 668 nm laser beam. (c) Exemplary data analysis. The graph above shows the optical power behind a drop of sample (*I*_s_) or water *I*_H_2_O_ and the reference power (*I*_ref_) measured simultaneously. The temperature rise (graph below) in the sample (*T*_s_) water colloid and the solvent itself (*T*_H2O_) must be known to evaluate the light-to-heat conversion efficiency. (d) Typical image of a droplet during heating; a scale is visible above the droplet—elements with a measured distance of 6.1 mm.

The iHCE (calculated as will be defined in chapter 2.3) was evaluated in a miniaturized setup,^32^ shown schematically in Figure 1a, which requires only approximately 10 μL of sample. Droplet (Figure 1b,d) volumes within different experiments were typically within the 12–15 μL range and were smaller (6–9 μL) if the surface tension of the sample was less than that of water. However, within one experiment, care was taken to ensure that the difference between the volume of the sample droplet and the volume of the water droplet (reference) was less than 0.5 μL. The photoexcitation beam spot diameter was approximately 1 mm^2^, and the optical path (droplet diameter) was up to 3 mm. The following continuous-wave laser diodes were used (all from Changchun New Industries Optoelectronics Technology Co., Ltd.): 400 nm (100 mW), 445 nm (1.5 W), 532 nm (1 W), 668 nm (1 W), 793 nm (3 W), 808 nm (2 W), 940 nm (2 W), 980 nm (10 W), and 1060 nm (2 W). The kinetic, time-resolved temperature profiles were registered by a thermographic camera (FLIR T540, accuracy ±0.5 °C with a reference, thermal sensitivity <40 mK, 24° @ 30 °C). Optical power behind the sample and the reference power were evaluated with two power meters (photodiode S120C head and PM100USB power meter, Thorlabs). The radiation power was 90 mW or less (above 40 mW) for wavelengths >900 nm to minimize overheating of the water in this range. Samples were diluted to obtain a temperature rise in the 1–5 °C range. The selected power is an experimentally determined optimum value (see Figure S1: power dependence of internal HCE and eHCE). The 120 mW and higher illumination power iHCEs are poorly reproducible (due to the dilution of the sample causing small and hard-to-determine absorbance value), while measurements at lower powers mean that the sample must be concentrated, which may affect the droplet density and result in the inability to maintain a stable droplet at the tip.

Measurements were conducted in a humidity chamber to minimize evaporation of the droplet. The humidity was found to be critically important to keep the volume of the droplet constant over ca. 8 min of experiment under laser illumination and heat generation. To prove it, we conducted an experiment in which we illuminated water drops of the same volume with a 980 nm laser beam at 90 mW for a time corresponding to standard measurements, but one measurement was carried out in a humidity chamber and the other in the external humidity conditions of the laboratory room (about 65%). In the first case, the droplet shrunk by less than 2%, while in the second case, the droplet volume was 7% lower at the end of the experiment. In addition, at low humidity, the temperature of the droplet (visualized by a thermal imaging camera) is lower than the ambient temperature, which is due to its evaporation and can lead to an incorrect temperature reading and ultimately to incorrect result evaluations (Figure S2).

The procedure of setup alignment and droplet formation has been described in our previous report.^32^ Briefly, the droplet was formed, positioned, and photographed on a contrasting background to precisely determine its size. Afterward, it was sealed inside a humidity chamber and left to stabilize its temperature for 5 min. Simultaneously, the laser was turned on to stabilize, but the beam was still blocked. Then, recordings by a thermographic camera and power meters were started. After 30 s, the laser beam was uncovered and the heating profile was registered during 2 min (Figure 1c). Then, the laser was turned off and the cooling curve profile was registered for up to 3 min. At the end of the recording of the thermal transient, an additional photograph was acquired to confirm the initial measurement of the droplet size.

#### 2.2.2 Data Analysis

The droplet size was determined from thermographic camera data in thermographic camera FLIR Tools software: the number of pixels forming a drop was determined by home-made software (i.e., height and variable diameter), and based on the scale bar (visible in the field of view), the real droplet volume was calculated. The temperature was averaged from the whole available droplet surface excluding edges (pixels in the temperature range between the real temperature of the droplet and the temperature of the background). Since the inherent thermographic camera accuracy is 2 °C, we made efforts to improve this value by concurrently taking the reference measurement of a background, which remained at a constant temperature. We have subtracted this reference value, captured on the same picture, to minimize artifacts originating from the electronic noise of the camera. As we have verified, this operation allowed one to increase the accuracy of the temperature measurement with the thermal imaging camera to less than about 0.5 °C. Data from FLIR Tools and Thorlabs optical power meters were exported and then analyzed in Origin 2019 software. A similar example of data analysis using Microsoft Excel and ImageJ software is shown in the Supporting file.

### 2.3 Methodology of Light-to-Heat Conversion Efficiency Evaluation

The calculations were performed based on the Wang model^20^ under the assumption that in a droplet system the effective mass of the system was the mass of the droplet (it has been shown that results obtained in this way correspond to the results of measurements in a cuvette with an independently determined effective mass).^32^ When the heating of the solvent is not negligible, it is necessary to subtract the associated term (*Q̇*_0_). If the heat from heating the solvent itself is not subtracted, the efficiency in that case could exceed 100%, since the denominator of eq 1 takes into account the absorbance, which is measured with a water reference. Because of this consideration, we have calculated the iHCE from the following equation
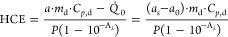
3The parameters *a*_0_ and *a*_s_ were both evaluated in the same way from the heating profile of the sample and the solvent, respectively, whereas the mass of the droplets of the dispersion or the solvent alone was considered equal. Care was taken to obtain the solvent droplets as close in size as possible to the sample drops.

The iHCE, by itself, does not give information on how much of the supplied energy was converted into heat (i.e., external conversion efficiency) but provides information on only how much of the absorbed energy has been converted into heat (i.e., internal conversion efficiency). Laser radiation can not only be absorbed but also be scattered, reflected, or refracted by the material. In laboratory practice, the extinction coefficient is used, which is the sum of the absorption and scattering coefficients
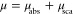
4If the scattering coefficient of the material has a large contribution, this introduces an additional error that results in a reduction of the calculated iHCE. The photons that are actually scattered could be erroneously included, while only the absorbed ones should be considered. Second, the light energy absorbed by some materials can be naturally emitted as photons. The presence of a finite value of photoluminescence quantum yield (PLQY) intrinsically limits the capability of the material to convert absorbed photons into heat. However, from a practical point of view, it is also necessary to consider the absorption properties to determine the required material concentration and to select the radiation dose. For this reason, we propose a new efficiency measure for NHs, external HCE, which takes into account both the iHCE determined so far and the mass absorption coefficient *a*, which allows for quantitative characterization of the material. eHCE represents how much of the incident pump power is transformed into heat.
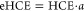
5The mass absorption coefficient can be determined from Lambert–Beer’s law written in the mass form
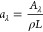
6where *A*_λ_ is the absorbance at a given wavelength, ρ is the mass concentration (mg/mL), and *L* is the optical path (cm). The absorbed pump power could be underestimated for highly scattering samples, which however we were avoiding by proper surface modifications (to form stable colloids). We chose the mass coefficient instead of the molar coefficient because in the case of nanoparticles, the accurate determination of the molar concentration is challenging due to the presence of a finite size distribution and possible inhomogeneity in the particle composition (as is the case for the Ag-Ag_2_S dimers herein studied). Moreover, when biomedical applications such as PTT are sought after, mass concentrations are usually preferred over molar ones.

## 3 Light-to-Heat Conversion Mechanisms

**Figure 2:**
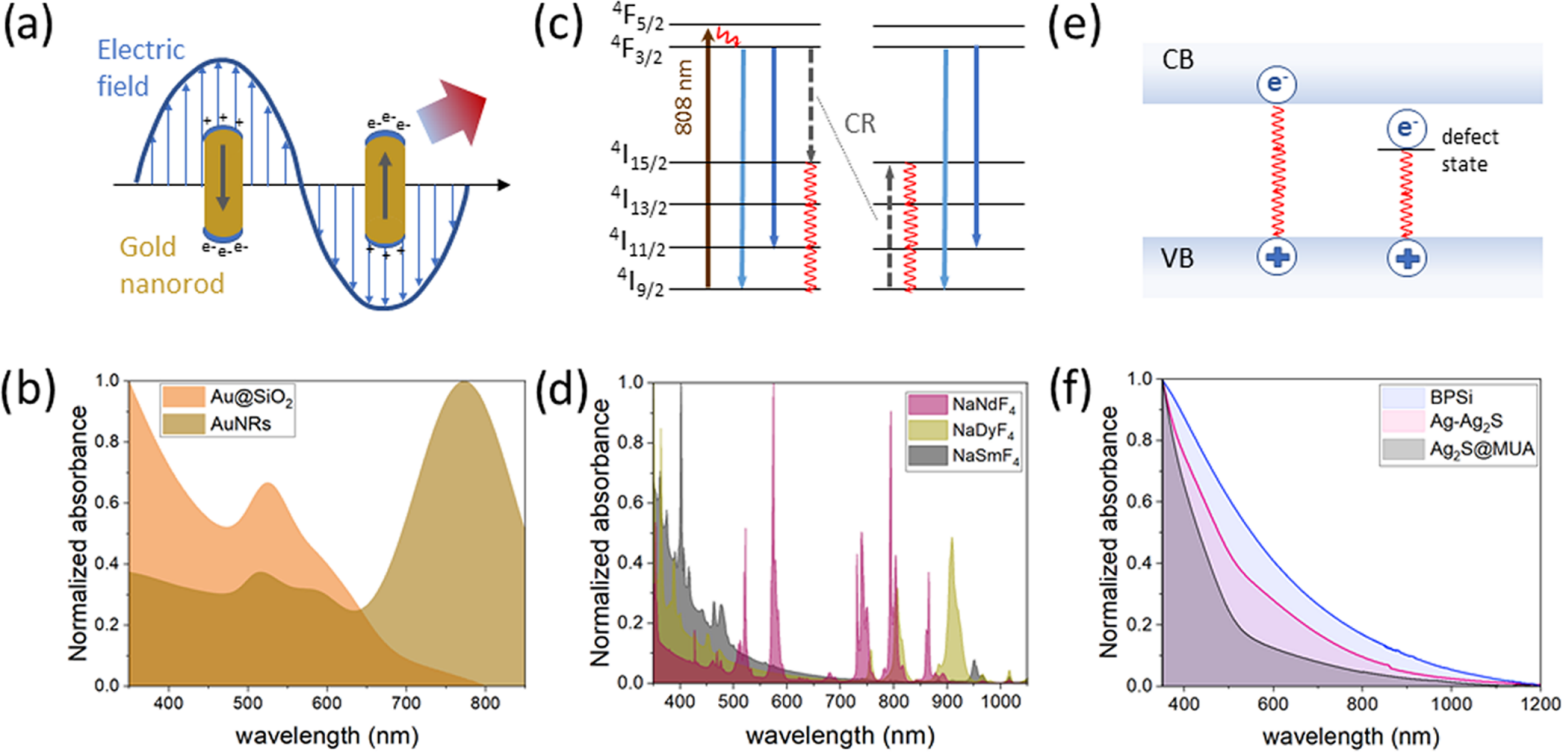
Mechanisms of heat generation and absorption spectra in different classes of NHs. (a) Localized surface plasmon resonance in plasmonic NHs. (b) Absorption spectra of gold nanospheres and gold nanorods. (c) Cross-relaxation in Nd^3+^ ions, which is the heat generation explanation for Nd^3+^ ions. (d) Absorption spectra of NaNdF_4_, NaDyF_4_, and NaSmF_4_ nanoparticles dispersed in chloroform. (e) Mechanism of heat generation in semiconductors. (f) Absorption spectra of semiconductor nanocrystals investigated in this study.

The most common mechanism of light-to-heat conversion relies on multiphonon relaxation of the excited states (Figure 2). After irradiation of the material, excitation from the ground state to the excited state occurs, which is followed by relaxation via internal conversion and vibrational relaxation to the lowest excited singlet state (for organic molecules), valence band (in semiconductors), or ground state (in lanthanides). This former mechanism may be preceded by singlet → triplet energy intersystem crossing or energy/charge exchange, which are competitive to radiative processes responsible for the reemission of the delivered energy in the form of photons. There is also a possibility of heat generation via the surface plasmon resonance process, which is typical for metallic NHs.

Plasmonic NHs are generally metallic nanomaterials that convert electromagnetic energy into heat through the surface plasmon resonance process. More specifically, heating is caused by a joule dissipation of oscillating electrons (Figure 2a). Surface plasmon resonance depends on the size and morphology of the nanoparticles.^18,20,33^ These differences are evident in the extinction spectra; for example, gold nanoparticles in the form of nanospheres have one absorption peak between 500 and 600 nm, whereas for larger nanoparticles, the maximum is red-shifted.^33^ Position of the peak far from NIR-I makes it of limited use in photothermal therapy. In contrast, gold nanorods have two absorption peaks (Figure 2b), one in a similar range as nanospheres, corresponding to the transverse mode, and a second, stronger absorption peak located in the near-infrared, corresponding to the longitudinal mode. Gold NHs in other shapes, such as nanostars, nanoshells, bipyramids, hexapods, and others, have also been designed to achieve absorption in a different range or to improve the iHCE.^34^ It was demonstrated that the efficiency is higher for nanorods than for nanostars^20^ and that the iHCE decreases with increasing nanoparticle size, which can be explained by the increased scattering of incident photons on these nanostructures. Lindley and Zhang have shown that smooth hollow gold nanospheres show a slightly higher efficiency than bumpy ones.^18^ In addition to gold and silver NHs, Cu_2–*x*_Ss,^22^ despite being semiconductors, also exhibit plasmonic properties due to free charge carriers. However, in this case, in place of free electrons in the conduction band, free vacancies occur at the top of the valence band. The plasmonic materials we have included in our study are AuNRs, CuS@GSH, and CuS@cit.

Lanthanide-ion-doped NHs have been primarily exploited for bioimaging and luminescence thermometry due to their rich energy-level diagrams, nonblinking and nonbleaching luminescence, long luminescence lifetimes, and narrowband absorption (Figure 2d) and emission, but they also show some promise for heat generation.^36−41^ Lanthanide-doped nanocrystals in general show high stability and low toxicity and can be easily functionalized;^36^ however, their absorption cross section is typically low, and because of that, it is debatable if they are suitable for PTT. Strategies to address the issues related to poor absorption cross sections by, e.g., conjugating them to “antennas” (plasmonic, dyes, etc.) are still under development.^42^ An important example of materials that meet the requirements necessary in biomedicine is NHs doped with neodymium ions.^37,38^ The advantageous feature of this ion is the location of its excitation band in NIR-I (i.e., ∼808 nm) and emission in both NIR-I and NIR-II (ca. 860, 1060, and 1300 nm). Pioneering work in the use of neodymium-doped nanomaterial not only as an excellent emitter but also as an NH was performed in 2010 by Bednarkiewicz et al.^37^ On the other hand, the first team to use neodymium for ex vivo studies was Rocha et al. in 2014.^39^ Since then, many attempts have been made to use rare-earth-doped nanocrystals as heaters, including studies on animals.^40,43−45^ In the case of the Nd^3+^ ion, the mechanism causing heat generation is the concentration quenching of {^4^F_3/2_, ^4^I_9/2_} ↔ {^4^I_15/2_, ^4^I_15/2_} through cross-relaxation transitions and through a subsequent series of nonradiative multiphonon depopulation steps of higher excited states (Figure 2c).^41^ This mechanism is probably responsible for the conversion of light to heat in the NaYF_4_-based materials we studied.

For semiconductor nanocrystals, heat generation is due to the nonradiative recombination of free electrons and holes (Figure 2e)^46^ and intraband nonradiative deexcitations. For NHs in the quantum dot regime, absorption and emission strictly depend on NH size, which is due to the quantum confinement.^47^ Even though quantum dots have a good long-term photostability and chemical stability,^23^ some of them display photoblinking and certain compositions can be cytotoxic.^10^ In this work, we study Ag_2_S, which is in the quantum dot regime. However, the biosafety risks could be reduced by surface passivation and its biofunctionalization^48^ or by selecting Pb- or Cd-free compositions (e.g., CuInS_2_, Ag_2_S, etc.). Among the semiconductors, porous silicon deserves special attention because silicon is commonly found in tissues as a trace element and is present in, e.g., drinking water; thus, it can be simply absorbed and excreted safely.^49^ Moreover, porous silicon has a large specific area and its surface could be easily biofunctionalized^41^ for drug loading and biotargeting.^50^ Herein, we included black porous silicon for which the proposed heat conversion mechanism is nonradiative carrier recombination.^51^

Carbon NHs are used primarily in solar energy applications. For photothermal therapy, carbon dots (CDs) deserve special attention due to their small size; moreover, CDs are characterized by their easy surface functionalization and good dispersibility and, importantly, for their biomedical applications, low toxicity, and good biocompatibility.^52^ Due to the numerous mobile π-electrons, strong electron–electron scattering and weak electron–phonon interactions occur. It was speculated that π-electrons act similarly to the free electrons in metallic nanoclusters rather than semiconductor QDs.^53^ However, CDs typically have absorption in the UV/vis range, and research is ongoing to develop techniques to shift the absorption toward the NIR. In our research, we included conventional CDs absorbing in UV and vis ranges.

Among the various metal oxide nanoparticles, those based on iron oxides are the most widely used for numerous applications. Iron oxide NHs are primarily known for their ability to generate heat under magnetic field stimulation, making them suitable for use in magnetic hyperthermia. However, these materials also exhibit high absorption capacities, making their use in photothermal therapy possible. Moreover, it has been shown that the therapy with optical excitation leads to better results for iron oxides, magnetite and maghemite.^54^ There have also been reports of successful combinations of these two therapies, which synergistically improved the effectiveness of the therapy.^30^ Iron oxide NHs are characterized by good biocompatibility and biodegradability.^55^ For these NHs, the heat generation mechanism is not yet well understood.^56^ Among the investigated NHs, the γ-Fe_2_O_3_-Au and γ-Fe_2_O_3_ belong to this group.

To quantitatively compare various colloidal nanoparticles for their suitability in PTT, we versatilely characterized their structural and spectroscopic properties and finally exploited a single optimized optical setup to measure iHCE.^32^ Herein, iHCE and eHCE results obtained for each material and their wavelength dependence are presented, and the best NHs are discussed.

## 4 Results and Discussion

### 4.1 Results by Materials

**Figure 3:**
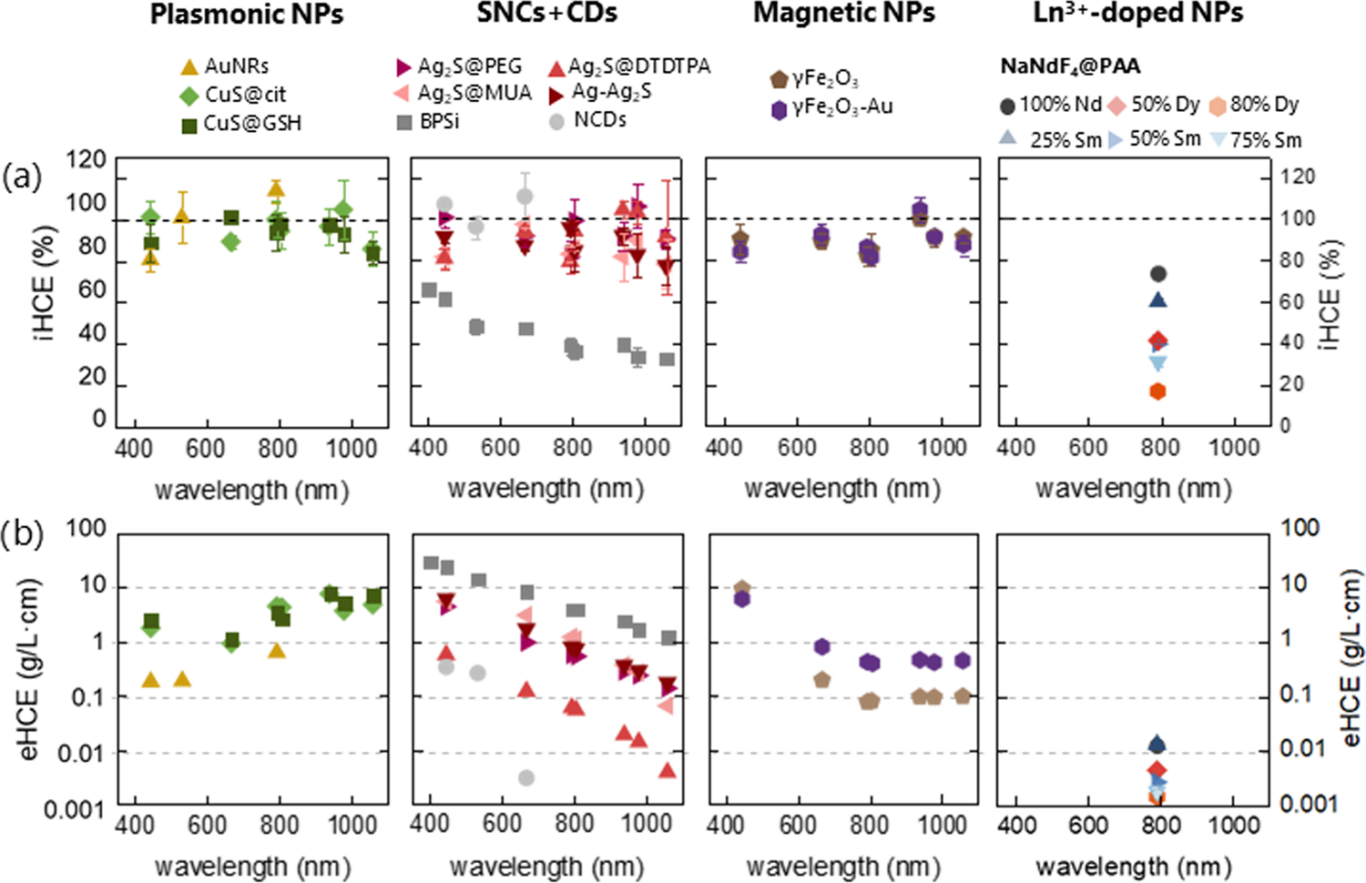
Ranking of the nanoheaters studied in this work. (a) Light-to-heat conversion efficiency as a function of wavelength. (b) External light-to-heat conversion efficiency as a function of wavelength.

Plasmonic NHs are one of the most well-studied classes of NHs. We have determined the iHCE of 28.8 nm × 8.0 nm gold nanorods to be close to 100% (Figure 3 and Table S1) at 794 nm, which is similar to 94% for 63.8 nm × 24.5 nm nanorods measured by Wang et al.^20^ AuNRs can therefore be regarded as one of the most effective light-to-heat-converting materials. Although for 532 and 794 nm the measured iHCE values exceed 100%, the material was stabilized by CTAB, which increased the viscosity of the sample and thus the droplet size needed to be reduced, which increased the measurement error. The different viscosity can affect the geometrical properties of the droplet, which translates into a change in optical properties and hence possibly introducing additional systematic error. To minimize this impact, the sample was diluted and a water droplet with a matching optical path was chosen as a reference.

For ca. 7–9 nm round-shaped CuS samples, which feature plasmonic properties similar to those of AuNRs, close to 100% iHCE was obtained. To understand whether the type of coating affects the iHCE, CuS coated with glutathione and citrate ions was studied at different excitation wavelengths. No significant difference between samples covered with glutathione and citrate was observed in the vis and NIR ranges, which is in agreement with the result obtained by Marin et al. at 806 nm.^22^ Although the exact iHCE values differ (94–100 vs 71%), the discrepancy may be caused by varying assumptions about the effective mass of the measurement system, as well as differences in the optical path, since in the case of drops the optical path is shorter and therefore the influence of scattering is reduced.^22^

In our research on lanthanide NHs, we focused on the Nd^3+^ ion due to its well-understood and easily controllable photophysical properties of nanomaterials based on such an ion. Although neodymium-containing materials have already been published by many authors, the obtained results in the efficiency of converting light to heat have either not been quantified or simply significantly differ between different studies, from 9^45^ to 85%^57^ (see Table S2). As shown in the literature, the efficiency can be influenced by the size of the nanoparticle as well as the thickness and the type of coating,^57^ and these also differ in various studies. The effect of coating is important because it functions as a thermal impedance.^58^ Our results show an iHCE of 74% for the NaNdF_4_ covered by PAA. To further progress with the understanding and optimization of Ln^3+^-doped nanoheaters, we hypothesized that adding dysprosium or samarium ions could increase the iHCE because these ions have a dense ladder of energy levels through which nonradiative relaxation could occur. To verify this hypothesis, we conducted a series of measurements for NaNdF_4_ materials doped with samarium and dysprosium ions (which were replacing Nd^3+^ ions in the pristine composition), coated with PAA for greater stability. As it turned out, the addition of a dopant did not have a positive effect: on the contrary, it caused a reduction in iHCE (Figure S3). The possible explanation is that the presence of samarium or dysprosium introduces additional energy diffusion that is unfavorable for heat generation, which occurs through cross-relaxation. Furthermore, by increasing the amount of Sm^3+^/Dy^3+^ ions replacing Nd^3+^, the Nd^3+^ ions are more distant from each other, reducing the possibility of cross-relaxation between them. Moreover, if the dopant replaces the dominant ion and does not absorb itself at the radiation wavelength, it reduces the absorption capacity of the NH designed in this way, which is undesirable.

Our results show that the attempt of adding a dopant instead of Nd^3+^ ions did not allow increasing the iHCE above 80%. This may partially explain the nonzero quantum yield, but it is not a complete explanation, as for highly doped NHs (25% Nd^3+^), it is less than 5%.^59^ Xu et al. recently showed that the iHCE decreases as the size of the nanoparticle increases (due to less surface quenching), and the effect is even more pronounced as the size increases after applying an inert coating due to better surface protection.^57^ However, the coating of the PAA material was necessary to obtain the time-stable and reproducible materials.

Ag_2_S nanoparticles are of increasing interest due to their large absorption cross section (3.46 × 10^–22^ cm^2^ at 800 nm) and iHCE of 93%, according to the literature (Ag_2_S with PEG coating).^60^ In our work, we have investigated ca. 3–9 nm dot-shaped Ag_2_S with different coatings: poly(ethylene glycol) (PEG), dithiolated diethylenetriamine pentaacetic acid (DTDTPA), mercaptoundecanoic acid (MUA), and Ag-Ag_2_S dimers. We have observed that in all of these NHs, the iHCE is higher than 75%. We observed that at 794 nm the excitation iHCE is the highest for Ag_2_S@MUA and the lowest for Ag_2_S@DTDTPA. This difference could be partially explained by PLQY (Table S3): for Ag_2_S@DTDPTA, the highest (0.66%) PLQY was observed. In addition, the coating can create impedance and induce differences between the temperature of the medium and the temperature of the nanoparticles;^58^ hence, it can differentially affect the iHCE.

In our comparison, we include a representative of silicon materials as well, i.e., BPSi, which has an irregular shape with a diameter of around 190 nm (Figure S4). Similar conversion efficiencies to those of Xu et al.^24^ at 806 nm (34% in the mentioned work and 36% on our measurement system) were obtained. We also proved that higher efficiencies could be obtained for shorter wavelengths, which suggests that this material can be useful in sunlight-based devices.^1^

To broaden the range of materials, we examined ca. 8 nm N-doped CDs. Their absorption (Figure S5) allowed us to measure iHCE only in the visible range. Our results have shown iHCE close to 100%. If the material does not exhibit luminescence and scatters poorly, a high (close to 100%) iHCE can be expected. Typically, the PLQY of CDs is on the order of a few percentage points^61^ unless special efforts (e.g., doping) are taken to improve it, so the preferable pathways are nonradiative relaxations. Due to the large discrepancy in the literature regarding the PLQY of CDs, and the observation that our CDs also show some luminescence, we performed a PLQY measurement (Figure S6) and obtained a PLQY of 3.2% for 445 nm excitation. This means that in this case it can be assumed that almost all of the absorbed light energy is converted into heat and the artifacts from scattering are practically absent. An example of a work in which red light was used to excite CD nanoparticles was demonstrated by Ge et al.,^62^ who obtained 38.5% iHCE and demonstrated in vivo studies on mice.^63^ Similarly, Geng et al. presented nitrogen and oxygen codoped CDs with 38.3% iHCE at 808 nm.^100^ Higher iHCE was obtained for supra-carbon nanodots: 52% for 732 nm and 53% for 808 nm.^101^

Our results for materials belonging to the iron oxide class show that both spherical γ-Fe_2_O_3_ and maghemite nanoflowers decorated with ultrasmall gold nanoparticles are characterized by high (>80%) iHCE. Diverse iHCEs for iron oxide NHs mentioned in the literature were obtained. Lozano-Pedraza et al.^56^ presented that for iron oxides with a dominant maghemite phase, the NH size (in the range of 9–18 nm) does not significantly influence the iHCE, but different shapes result in different iHCEs. In contrast, Sadat et al.^64^ showed a graph presenting the size dependence of different Fe_2_O_3_ NHs, where the iHCE decreased with the rise in NH size from ∼80% iHCE for 10 nm NHs covered by PAA to ∼30% for ∼100 nm NHs covered by PS to ∼18% for Fe_2_O_3_ beads.

A direct comparison of iHCE values with literature values is difficult because, first, iHCE is not always determined as an exact value and, second, when it is determined, it is often influenced by factors such as the position of the temperature sensor and assumptions considering the mass of the sample.^32^ Furthermore, previous literature results were often obtained for other wavelengths and for materials that differ in morphology.

### 4.2 Selection of Therapeutic Wavelength

It is preferable to perform PTT with absorption wavelengths falling into the biological windows. The reason for this choice stems from the lower absorption coefficient of water and of other components of biological tissues. Moreover, reduced light scattering occurs at longer wavelengths. Doing so, the light penetration depth can be extended significantly as compared to that achievable with shorter wavelengths. Usually, the excitation wavelengths are selected also depending on the NH absorption maxima because by using such wavelengths the greatest part of the excitation light can be absorbed. This is critically important for PTT because increasing the photoexcitation intensity is not possible beyond the permitted light dose exposure for a given tissue or skin; typically, under normal conditions, a maximum excitation power density of 330 mW/cm^2^ is commonly accepted for tissue examination, but values of up to 2 W/cm^2^ are used for PTT.^9^ However, apart from the absorption capacity, it is also necessary to know the iHCE, which, as we show for some materials, strongly depends on the wavelength (Figure 3a).

The determination of such a correlation was only possible for materials with a broad absorption band. Although only NIR is primarily used for therapeutic purposes, knowing how efficiently photons of different energies are converted to heat can be useful also to understand the mechanisms of light-to-heat conversion. The most glaring example is BPSi, for which the iHCE is the highest at shorter wavelengths and follows a similar trend of the absorption spectrum. Thus, although the material converts absorbed ∼800 nm photons with iHCE close to 35%, for visible light, the iHCE is almost doubled while the absorption capability also increases. A similar effect was observed for silicon nanoparticles by Regli et al.^51^ In that work, 488, 514, and 647 nm photoexcitation wavelengths were investigated and the iHCE decreased from 64 to 51%, which was explained by carrier thermalization contribution to photothermal effects. They also observed that photoluminescence intensity in this spectral range is higher at a longer wavelength; however, in the case of BPSi, no luminescence was observed.

In contrast, for the CuS, the iHCE as a function of wavelength does not change significantly. For this NH particle type, we can assume that the quantum yield is close to zero at all explored wavelengths. Similarly, for Ag_2_S, Ag-Ag_2_S dimers, AuNRs, γ-Fe_2_O_3_, and γ-Fe_2_O_3_-Au, no obvious wavelength dependence was observed. Different results were presented on the work on maghemite,^56^ which showed wavelength dependence in the range of 700–1280 nm, and the iHCE almost doubled at the longest wavelength; however, these results were obtained for a material with different morphology and size. There are very few publications illustrating iHCE as a function of wavelength, while for materials that show no luminescence and where the impact of scattering on the results is negligible, we expect a constant value of iHCE.

### 4.3 Selection of the Most Effective Materials and Further Perspectives

**Figure 4:**
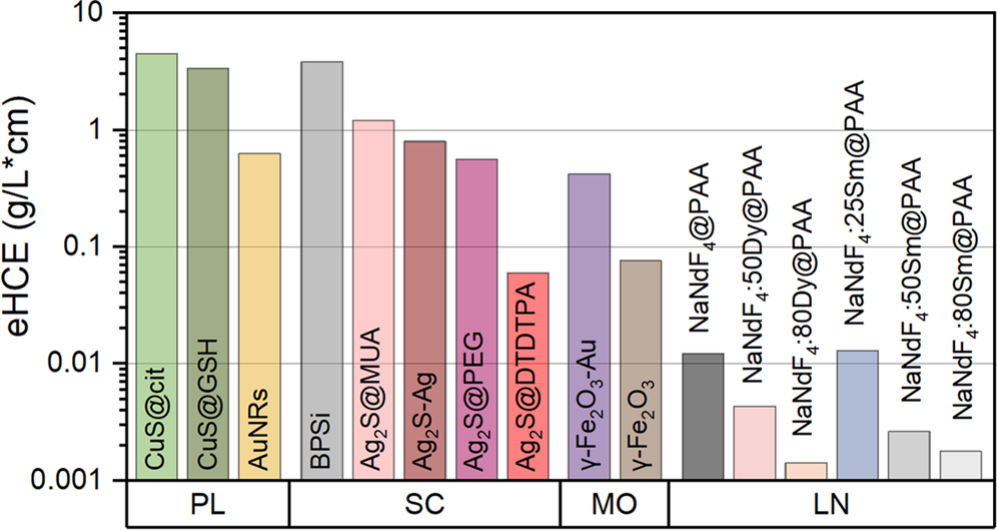
External light-to-heat conversion efficiency at 794 nm: ranking of nanoheaters investigated in this study.

Selection of the best material is not a trivial task. Although Figure 3a shows that for most materials there is no wavelength dependence of iHCE, this is not always the case and providing the iHCE value requires specifying at which wavelength the value was determined. Moreover, because of the wavelength dependence of the absorption coefficient, eHCE is also strongly affected by the wavelength (Figure 3b). In Figure 4, we decided to limit the presentation of the iHCE and eHCE to 794 nm, which is generally considered an optimal wavelength for PTT. The numerical data are given in Table S1.

From the eHCE results (Figure 3b), it can be seen that the vast majority of the materials studied shows the best properties for shorter wavelengths, in the visible range. This observation defines the first challenge for NHs dedicated to PTT, namely, to design new materials that provide a sufficiently high absorption coefficient for the NIR-I. Furthermore, as the wavelength increases, the absorption of water also increases, making heating no longer selective. The evidence for this may be the heating of a water droplet at the same power, which is negligible in the first optical window and significant above 900 nm (Figure S7). This obstacle defined the next challenge to search for NHs absorbing efficiently in the optical windows, e.g., at 800 nm. The importance of the absorption coefficient is clearly seen for BPSi, which, despite its relatively low iHCE, has the highest eHCE coefficient in the vis range. The probable reason for such outstanding absorption capacities is the porous structure of the material, which translates into its relatively lower mass at a similar volume. The highest eHCEs at NIR-I and NIR-II are observed for CuS. The reason for the increasing eHCE at longer wavelengths is the position of the maximum of absorption at 926/1037 nm (citrate/GSH coating). Ag_2_S and Ag-Ag_2_S dimers also reach high eHCEs in NIR-I. In the case of Ag_2_S, the eHCE strongly depends on the coating; the value found for the DTDTPA coating is one order of magnitude lower than for MUA and PEG, and the highest values are observed for MUA. The discrepancies in the values observed for the different Ag_2_S might stem from the following reasons: different size (hence different surface-to-volume ratio), different vibrational energy of the attached molecules, and different relative weight of the ligand shell vs the total weight of the sample (including the inorganic Ag_2_S core and the ligand shell). The high eHCE of γ-Fe_2_O_3_-Au (0.420 L/(g·cm)) with regard to γ-Fe_2_O_3_ (0.076 L/(g·cm)) could be explained by the higher intensity of plasmonic coupling between the gold nanoparticles. For instance, it was reported that the optical specific absorption rate (SAR) values (see^30^) increase from 2940 W/g for γ-Fe_2_O_3_ up to 4581 W/g in γ-Fe_2_O_3_-Au,^65^ and the value can be further controlled by linkers. In the present case, indeed, the plasmonic effect in gold nanoparticles enhanced the heat generation by enhancing the absorption capacity. The photothermal conversion depends on several material parameters such as size, shape, surface, aggregation status, etc., which can affect light absorption and scattering properties, which in turn can affect the heat generation or conversion efficiency. This creates additional difficulties in quantitatively comparing materials with different properties. Our intention was to select NHs with a variety of compositions and morphologies to show a comparison of as many materials as possible on the standardized experimental system. However, this is only a fraction of the numerous NHs already known.^1,7,12,41^ We also speculate that many of the materials presented in numerous publications can achieve high iHCE scores and even higher eHCE values than the ones presented in this work. Moreover, the approach and methodology described in this work can also be used to compare other materials, including organic materials such as supramolecular assemblies,^66,67^ amino acids,^68^ and dyes, which are used in clinics (e.g., ICG;^14^ or next-generation dyes, e.g., croconium dye^15^). In this case, scattering is unlikely to have a significant impact on iHCE. The only limitations of the proposed method relate to photobleaching, which makes it impossible to record a temperature rise curve consistent with the model, as well as the solvents’ wetting angle must allow the forming of a droplet.

The proposed ways of comparing the NHs and the observed relative trends are envisaged to inform the design of NHs with the potential to be translated to the bedside as they allow us to unambiguously rank all of those different materials. Such a ranking should be supplemented in the future by studies of cytotoxicity, clearance kinetics, and biological interaction studies so that a reliable and versatile comparison of various NHs can help select the most appropriate candidate nanoheaters for PTT in vivo.

## 5 Conclusions

Advanced inorganic nanomaterials belonging to five different classes of materials were synthesized, and their iHCE values were measured using optimized and standardized experimental setups (with different laser diode light sources of the same light beam output). Most of the studied nanomaterials were found to display a high (>75%) light-to-heat conversion efficiency, which can be easily related to the low or absent luminescence. No significant effect of the laser wavelength on the iHCE was observed if the sample did not show scattering. Although one may claim that scattering leads to an underestimation of the iHCE, such materials will be less suitable for photothermal therapy due to the high risk of healthy tissues overheating and their lower absorptivity.

In contrast to expectations, for the NaNdF_4_ nanoparticles codoped with rare earth ions (Sm^3+^, Dy^3+^), we found that the cross-relaxations and multiphonon relaxations within the Nd^3+^ network are nevertheless more efficient that quenching at those additional ions. For this class of materials, increasing their suitability for photothermal therapy by improving their absorption cross section still remains a challenge. The progress in augmenting the absorption in combination with the feasible single bifunctional (heating and local thermometry using core–shell compositional architectures) lanthanide-doped nanoparticles promises further development of functional PTT nanoplatforms.

Although much attention has been given to the determination of the iHCE of NHs, from the perspective of ranking different materials, it should be complemented by knowledge of the mass absorption coefficient because iHCE alone is not sufficient to select the optimal dose of material and optical power. Of all of the materials measured, BPSi has the best absorption capacity in the VIS spectral range, while in the NIR range, the most suitable for PTT, CuS shows the most promising properties, and their application would require the lowest NH and irradiation dose.

## Supporting Information Available

List of chemical reagents; description of syntheses; power dependence of internal HCE and eHCE; thermal images of droplets at/without increased humidity conditions; calculated iHCE and eHCE of measured samples at 794 nm; HCE of Nd^3+^-doped nanomaterials (literature results’ comparison); iHCE of NaNdF_4_ as a function of the Sm^3+^/Dy^3+^ dopant concentration; emission QY under 808 nm CW laser irradiation at distinct laser power densities; morphology of the NHs; absorption spectra of CDs, NaNdF_4_ doped with Dy^3+^ and Sm^3+^, Ag_2_S, and γ-Fe_2_O_3_; QY measurement of CDs; temperature rise curves of a 13 μL water droplet for different wavelengths measured at constant irradiance power (90 mW); XRDs of the NHs; and a tutorial of data analysis using excel and ImageJ software (PDF)
Example analysis solver (XLSX)
