# Correction to “Quantitative Comparison of the Light-to-Heat Conversion Efficiency in Nanomaterials Suitable for Photothermal Therapy”

**DOI:** 10.1021/acsami.2c13851

**Published:** 2022-08-16

**Authors:** Agnieszka Paściak, Riccardo Marin, Lise Abiven, Aleksandra Pilch-Wróbel, Małgorzata Misiak, Wujun Xu, Katarzyna Prorok, Oleksii Bezkrovnyi, Łukasz Marciniak, Corinne Chaneac, Florence Gazeau, Rana Bazzi, Stéphane Roux, Bruno Viana, Vesa-Pekka Lehto, Daniel Jaque, Artur Bednarkiewicz

In the original version of the article the eHCE unit was mistakenly assigned as (g/L·cm) instead of L/(g·cm) in the figures (Figure 3, Figure 4, and TOC). The authors apologize for any confusion this may have caused. This correction does not alter any conclusions of this work, as in the text of the publication and in the Supporting Information the eHCE unit was given correctly.

**Figure 3:**
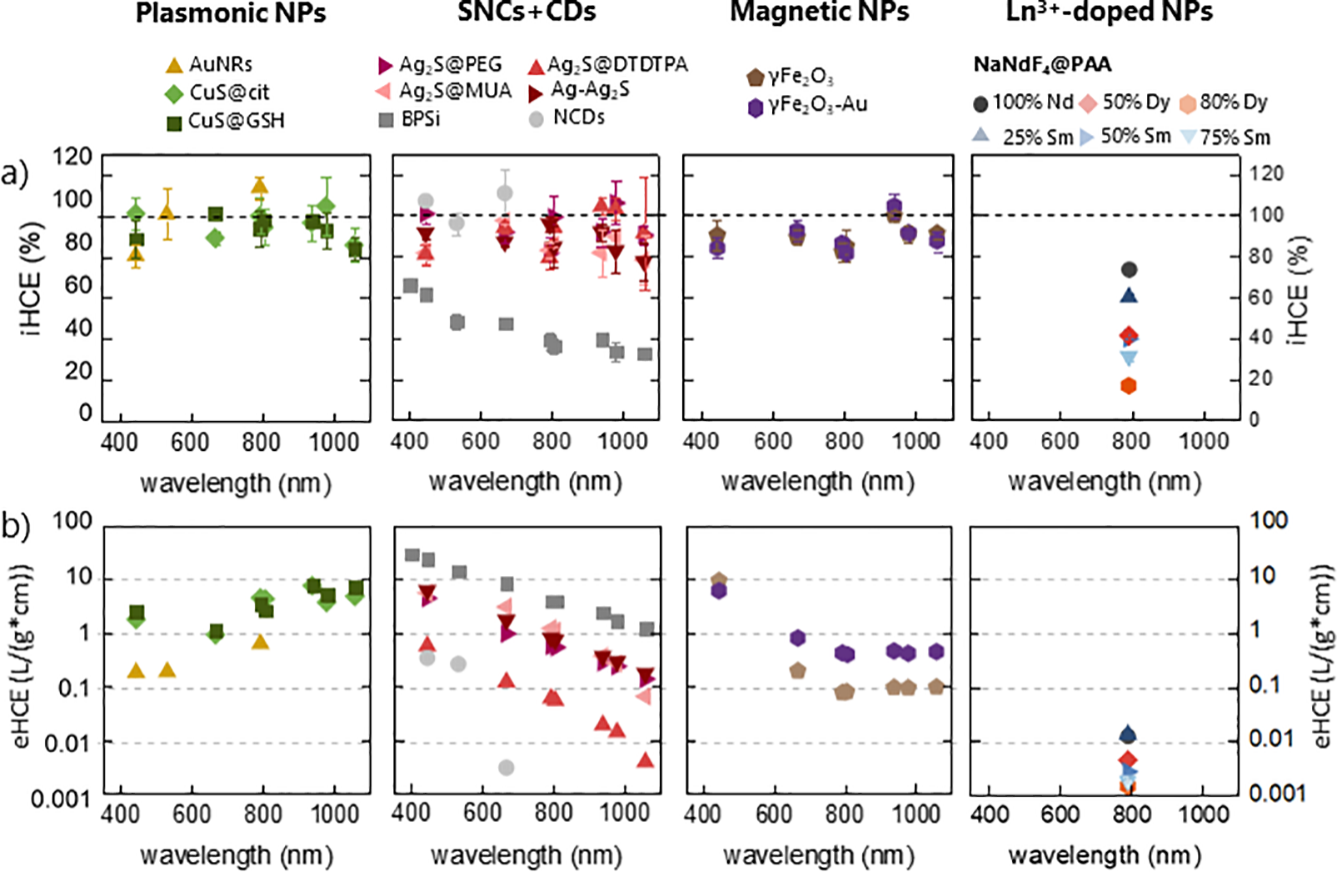
Ranking of the nanoheaters studied in this work. (a) Light-to-heat conversion efficiency as a function of wavelength. (b) External light-to-heat conversion efficiency as a function of wavelength.

**Figure 4:**
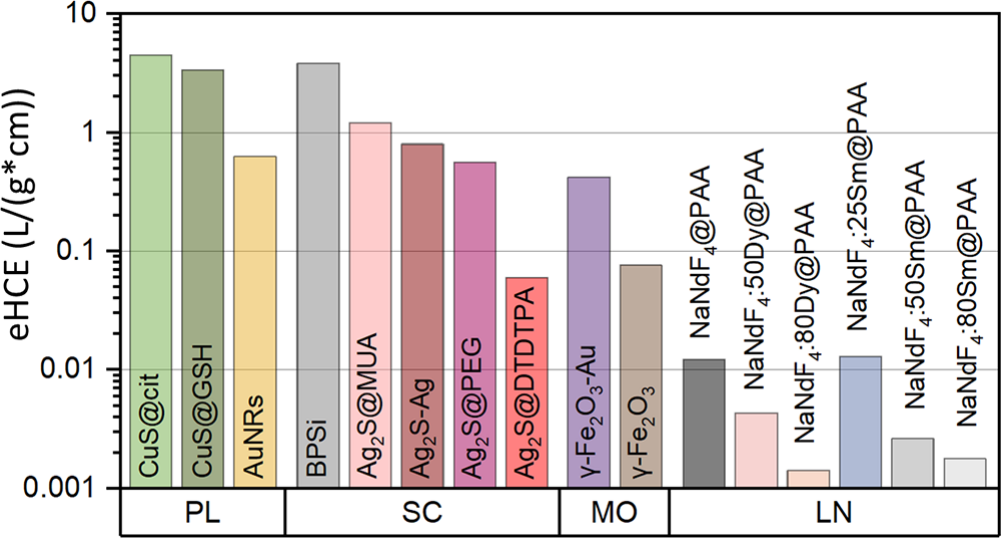
External light-to-heat conversion efficiency at 794 nm: ranking of nanoheaters investigated in this study.



