# Supplementary material for: Quantitative Comparison of the Light-to-Heat Conversion Efficiency in Nanomaterials Suitable for Photothermal Therapy
Source: ACS Appl Mater Interfaces. 2022 Jul 18;14(29):33555–66. doi: 10.1021/acsami.2c08013 (PMC9335407; doi:10.1021/acsami.2c08013)
Supplement: Supplementary file 1 — am2c08013_si_001.pdf [file am2c08013_si_001.pdf]

Supporting Information

Quantitative comparison of the light-to-heat conversion efficiency in nanomaterials suitable for photothermal therapy

*Agnieszka Paściak, Riccardo Marin, Lise Abiven, Aleksandra Pilch-Wróbel, Małgorzata Misiak, Wujun Xu, Katarzyna Prorok, Oleksii Bezkrovnyi, Łukasz Marciniak, Corinne Chaneac, Florence Gazeau, Rana Bazzi, Stéphane Roux, Bruno Viana, Vesa-Pekka Lehto, Daniel Jaque, Artur Bednarkiewicz**

*E-mail: a.bednarkiewicz@intibs.pl

List of figures and contents

Description S1 Chemical reagents 2

Description S2. Synthesis procedures 2

2.1.1 Synthesis of gold nanorods 2

2.1.2. CuS 3

2.1.3. $NaY_{1-x}Ln_xF_4$ 4

2.1.4. Ag-Ag_2S dimers 5

2.1.5. Ag_2S 5

2.1.6. BPSi 5

2.1.7. γFe_2O_3 6

2.1.8. γ Fe_2O_3 decorated with Au 6

2.1.9. CDs 7

Figure S1. Power dependence of internal HCE and eHCE. 8

Figure S2. Thermal images of droplets registered at increased and ambient humidity conditions. 8

Table S1. Comparison of the possibilities of using different materials in photothermal therapy. Data provided for 794 nm excitation. 9

Table S2 iHCE of Nd^{3+} doped nanomaterials 10

Figure S3. Light-to-heat conversion efficiency of $NaNdF_4$ in function of Sm^{3+}/Dy^{3+} dopant concentration. 10

Table S3. Emission quantum yield under 808 nm CW laser irradiation at distinct laser power densities.. 11

Figure S4 Morphology of the materials compared in this work 11

Figure S5. Absorption spectra of a) CQDs, b) NaNdF4 doped with Dy^{3+} and Sm^{3+}, c) Ag_2S, d) γ-Fe_2O_3 12

Figure S6. Photoluminescence quantum yield (QY) measurement of CDs. 13

Figure S7. Temperature rise curves of a 13 uL water droplet for different wavelengths measured at constant irradiance power (90 mW). 14

Figure S8 XRD of $NaNdF_4$ samples doped with Dy^{3+} and $Sm^{3+.}$ 15
Figure S9 XRD of CuS@GSH, CuS@cit and Ag-Ag_2S samples 15
Figure S10 XRD of BPSi, CDs and AuNRs samples 16
Figure S11 XRD of Ag_2S samples. 16
Figure S12 XRD of γ-Fe_2O_3 sample 16

Description S1 Chemical reagents

All of the chemical reagents in this experiment were used as received without further purification. Hexadecyltrimethylammonium bromide (CTAB, 99.0%), ammonium bromide (NH_4Br), sodium bromide (NaBr), hydrogen tetrachloroaurate trihydrate ($HAuCl_4·3H_2O$, 99.9%), silver nitrate ($AgNO_3$, 99.9%), iron(III) chloride hexahydrate ($FeCl_3·6H_2O$, 98%), iron(II) chloride tetrahydrate ($FeCl_2·4H_2O$, > 98%), sodium sulfide nonahydrate ($Na_2S·9H_2O$, 99.99%), 11-mercaptoundecanoic acid (95%), poly(ethylene glycol) methyl ether thiol (6kDa), L-ascorbic acid (99%), sodium borohydride ($NaBH_4$, 99%), samarium oxide (99.99%), neodymium oxide (99.99%), dysprosium oxide (99.99%), acetic acid (99%), diethylene glycol (99%), N-Methyldiethanolamine (NMDEA, ≥99%), pure oleic acid, citric acid (99%), sodium hydroxide (NaOH, ≥ 98%), and 1-octadecene (90%) were purchased from Sigma Aldrich Chemistry. Copper chloride dihydrate ($CuCl_2·2H_2O$, 99%), glutathione reduced (GSH, 98+%), sodium sulfide nonahydrate ($Na_2S·9H_2O$, 98%) were purchased from Alfa Aesar. Silver nitrate ($AgNO_3$, 99.85%) and trisodium citrate (Na_3(cit), 98% were purchased from Acros Organics. Sodium silicide (NaSi) was provided by SiGNa Chemistry Inc. HCl and HF were bought from Merck, $HClO_4$ (65%) from Carlo Erba, ethanol (96% pure p.a.), n-hexane (95%), acetone (pure p.a.), and chloroform were purchased from POCH S.A (Poland). Urea (pure p.a.) was purchased from Chempur (Poland). Isopropyl alcohol was purchased from Fisher, roasted coffee powder was purchased from Hacendado, Mercadona. DTDTPA is a non commercial product and was prepared as previously reported [1].

Description S2. Synthesis procedures

2.1.1 Synthesis of gold nanorods

Gold nanorods (AuNRs) were synthesized by a modified seed-mediated method [2]. Au seeds are small nuclei made of gold, which serve as the starting point for the development of a more complex structure. The synthesis was performed in a water bath at 30 °C.

Seed solution: 25 μL of 50 mM $HAuCl_4$ was added to 4.7 mL of 0.1M CTAB solution, the mixture was slowly stirred until a completely clear solution was obtained (about 5 min). Then, 300 μL of 10 mM $NaBH_4$ (freshly prepared) was added under vigorous stirring. The color of the solution changed from yellow to brownish yellow. The seed solution was stored at 30 °C (gently stirred). Obtained seeds were used for the synthesis of single crystal Au nanorods.

The growth solution was prepared by adding 100 μL of 50 mM $HAuCl_4$ to 10 mL of 100 mM CTAB solution. The mixture was kept for 10 min in water bath (30°C) to ensure complexation between gold salt and CTAB. Then, 75 μL of 100 mM ascorbic acid was added to the mixture, which was gently stirred (the solution became colorless). To the grown solution, 80 μL of 5 mM $AgNO_3$ was added. Finally, 120 μL seeds solution was added to the mixture. Obtained solution was vigorously stirred and the left undisturbed at 30°C for 30 min in water bath. Next, the solution of as-prepared nanorods was centrifuged at 8000 rpm for 30 min. The precipitate containing nanorods was dissolved in 3 mL hot (50 ºC) of 300 mM CTAB solution and transferred into a glass tube. Upon cooling at room temperature, a brown precipitate at the tube bottom was observed. The precipitate containing nanorods was separated carefully from the supernatant, finally dispersed in water (5 ml) and stored at room temperature.

2.1.2. CuS

The synthesis of copper sulfide (CuS) nanoparticles was performed slightly modifying the reported procedure [3]. In brief, 1 mL of a 0.1 M aqueous solution of $Na_2S{\cdot}9H_2O$ was swiftly injected into 20 mL of an aqueous solution of the selected ligand (trisodium citrate, Na_3cit, 0.06 mmol, 17.6 mg; L-glutathione, GSH, 0.1 mmol, 30.7 mg) and $CuCl_2{\cdot}2H_2O$ (0.1 mmol, 17 mg) in a 50-mL round-bottomed flask. After 5 min of stirring at room temperature, the flask was transferred to an oil bath pre-heated at 90 °C and the mixture was kept under stirring at that temperature for a preselected amount of time (30 min for Na_3cit and 20 min for GSH). Subsequently, the reaction mixture was quickly cooled down to room temperature in a cold-water bath and transferred to a 20-mL vial. For TEM observations, the particles were precipitated with isopropanol (iPrOH), recovered by means of centrifugation (30,000 g for 20 min at 4 °C), and washed once with a mixture of water and iPrOH, before being redispersed in water. The sample was stored at 8 ºC for further use. Assuming 100% synthesis efficiency, the estimated material concentration was approximately 0.5 mg/mL (disregarding the contribution from the ligands).

2.1.3. $NaY_{1-x}Ln_xF_4$

Preparation of precursor: The 2 mmol of acetate precursors ($(CH_3COO)_3Ln$) were prepared by mixing lanthanide oxides (Nd_2O_3, Sm_2O_3, Dy_2O_3 — 1 mmol) with 50% aqueous acetic acid. Prepared mixture was heated to 200 °C for 120 min in Teflon-lined autoclave. The final precursor was obtained by evaporation of solvents in vacuum and further drying (130 °C for 12 h).

Preparation of differently doped $NaYF_4$ material: The synthesis was analogous to procedure described before [4]. The given amount (2 mmol Ln^{3+}) of $(CH_3COO)_3Ln$ precursors were mixed in three-neck flask with oleic acid (12 mL) and octadecene (30 mL). The solution was stirred and heated to 140 ^{o}C under vacuum for 30 min to form a $Ln(oleate)_3$ complex and remove oxygen and water. Subsequently, the reaction temperature was decreased to 50 °C, and 8 mmol ammonium fluoride (NH_4F) and 5 mmol sodium hydroxide (NaOH) dissolved in 20 mL of methanol were added to the reaction flask. The reaction atmosphere was changed to nitrogen and maintained till the end of the synthesis. After that, the temperature was increased to 80 °C to evaporate methanol. This process took approximately 30 min. Finally, the reaction temperature was quickly increased to 300 °C and the solution was stirred at this temperature for 60 min. After the synthesis, the solution was cooled to room temperature. The obtained nanoparticles were precipitated with an excess of ethanol and centrifuged (18 000 g for 10 min). The obtained pellet was purified by washing with *n*-hexane and ethanol. The final product was dispersed in 5 mL of chloroform. Based on the synthesis conditions, assuming 100% synthesis efficiency the estimated material concentration was 20 mg/mL.

Polyacrylic acid (PAA) coating: To obtain water-dispersible samples, a ligand exchange method was applied. First, to remove the oleate ligands from the NP surface, 10 mg of NPs was transferred to an Eppendorf tube and centrifuged (20 min, 20000 g). The obtained pellet was redispersed in 500 μL of n-hexane. The mixture was sonicated as long as the pellet had been suspended. After that, 400 μL of acetonitrile and 100 μL of $NOBF_4$ dissolved in acetonitrile (0.16 M) were added to the tube, and solution was mixed for 10 minutes. In the next step the oleic stripped nanoparticles were precipitated by adding 500 μL of toluene to the Eppendorf tube and centrifuged. Finally, the BF_4^- capped nanoparticles were dispersed in 100 μL dry DMF. Next, 64 μL of PAA solution in water (80 mg/mL) was added to the reaction tube, and vigorously stirred for 20 minutes. After this time, and centrifugation (14 000 g, 15 min) the obtained pellet was dispersed in 500 μL of distilled water.

2.1.4. Ag-Ag_2S dimers

The synthesis of the Ag-Ag_2S dimers was performed according to the procedure reported in [5]. First, an aqueous coffee extract was prepared soaking 15 g of a roasted coffee powder (Hacendado, Mercadona, Spain) in 100 mL of de-ionized water for 2 h, followed by filtration, and dilution to a final volume of 120 mL. Subsequently, water (6.5 mL), coffee extract (3.5 mL), and a magnetic stir bar were added to a 20-mL vial. The initial pH of the solution was 5.5 and was later adjusted to 9.0-9.5 by addition of NaOH 0.5 M. In the meantime, $AgNO_3$ (0.1 mmol, 17 mg) was dissolved in deionized water (1 mL) and swiftly added to the diluted coffee extract. Upon addition of the Ag^+ solution, Ag NPs started forming immediately (as denoted by a marked darkening of the mixture) and the pH dropped. The pH was quickly readjusted between 9.0-9.5 with $NaOH_{aq}$ 0.5 M, and then 0.5 mL of a 0.1 M Na_2S solution in water was swiftly introduced in the reaction mixture. The color shifted almost immediately from dark orange to dark brown, indicating the formation of Ag_2S. Stirring was continued for 2 min and finally the reaction mixture was quenched in 15 mL of iPrOH. After centrifugation at 3,820 g for 20 min, the reaction product was collected as a pellet and the slightly orange supernatant discarded. Finally, the dispersion was filtered through a 200-nm cellulose filter and stored at 8 ºC for further use.

2.1.5. Ag_2S

Ag_2S nanocrystals were prepared using a hydrothermal method assisted by microwave heating [6]. Crystallization of silver sulphide from silver nitrate ($AgNO_3$, 5 mM, 2 eq.) and sodium sulphide ($Na_2S{\cdot}9H_2O$, 1eq.) in water solution at pH 8, is induced on a monomodal microwave cavity at 100 °C (300 W) during 5 min under magnetic stirring. Nanoparticle growth is controled by adding complexing ligands (CL) in the previous reactant's water solution before heating. Three different ligands have been used for this study, mercaptoundecanoic acid (MUA), polyethylene glycol (PEG) and dithiolated diethylenetriamine pentaacetic acid (DTDTPA) with a stoichiometry of 3 eq., 2 eq. and 1.5 eq. per silver atom respectively. After the microwave heating program, Ag_2S nanoparticles decorated with CL (Ag_2S@CL) were washed with absolute ethanol and finally, dispersed in water.

2.1.6. BPSi

BPSi was prepared according to a previously published protocol [7,8]. The reactants of NaSi, ammonium salt (NH_4Br), and NaBr were mixed with the mass ratio of 1:4:4. Then, they reacted in a tube oven under a N_2 atmosphere at 240 °C for 5 h. After cooling to room temperature, the raw product was washed with 2.0 M HCl and then further purified with 1% HF solution. The obtained product was washed with H_2O and ball-milled in ethanol (1000 rpm, 30 min). The

BPSi NHs with desired mean diameter around 200 nm were obtained via the removal of big particles with 1500 RCF centrifugation for 5 min.

2.1.7. γFe_2O_3

Maghemite, γFe_2O_3, were synthesized by coprecipitation of iron(II) and iron (III) cations as previously reported [9]. Magnetite spinel iron oxide Fe_3O_4 is first precipitated by addition of aqueous solution of ferrous and ferric ions (Fe(II)/Fe(III)=0.5) at pH 9 using a METROHM 3D Combititreur device. All solutions were carefully deaerated with nitrogen, which was continuously bubbled during the precipitation. After 1 hour of ageing, magnetite is centrifugated during 15 min at 8000 rpm and then, a perchloric acid solution (3 M) is added to magnetite for a fully oxidation step into maghemite γ-Fe_2O_3 [10]. The estimated obtained concentration is 42 mg/mL.

2.1.8. γ Fe_2O_3 decorated with Au

The synthesis of spinel iron oxide nanoflowers decorated with gold nanoparticles have been synthesized as previously reported [11]. Briefly, γ-Fe_2O_3 NFs were obtained from coprecipitation of $FeCl_3 \cdot 6H_2O$ (2.164 g; 8 mmol) and $FeCl_2 \cdot 4H_2O$ (0.796 g; 4 mmol) in 75 mL of diethylene glycol (DEG). After 1 hour of stirring, 75 mL of a solution of NMDEA is first added to the mixture and then, 80 mL of an alkaline solution of both DEG and NMDEA (1:1, v/v). The resulting mixture was stirred for 3 h, and then heated during 4 h at 220 °C. The black solid is washed with a mixture of ethanol, ethyl acetate and nitric acid (10%). At this stage, 20 mL of $Fe(NO_3)_3 \cdot 9H_2O$ ($4.951.10^{-3}$ mol) is added to the nanoparticles. The mixture is heated to 80 °C for 45 min to achieve a complete oxidation of magnetite in maghemite. After nitric acid treatment and washing with acetone and diethyl ether, γ-Fe_2O_3 NFs are redispersed in water.

In same time, gold nanoparticles stabilized with DTDTPA were synthesised by reduction of $HAuCl_4 \cdot 3H_2O$ ($5.1 \cdot 10^{-6}$ mol) in DTDTPA ($5.1 \cdot 10^{-6}$ mol) solution with an excess of $NaBH_4$ as described by Alric et al. [12]. After 1 h of vigourous stirring, 5 mL of aqueous hydrochloric acid solution (1 M) were added. Gold@DTDTPA nanoparticles were then precipitated and washed thoroughly and successively with 0.01 M HCl, water and diethyl ether. The resulting black powder was dispersed in 10 mL of 0.01 M NaOH solution.

Finally, as-prepared suspension of gold@DTDTPA are modified by dopamine and mixed with the suspension of maghemite nanoflowers (γ-Fe_2O_3 NFs, 6 mL, 35 g Fe/L). The mixture with a pH of 5.5 was heated at 50 °C for 24 h. After washing with ultrapure water, acetone, and diethyl

ether, an aqueous suspension of γ-Fe_2O_3 NFs-Au is obtained with an iron and gold concentration of 25 and 5 mg/mL respectively.

2.1.9. CDs

N-doped carbon dots were synthesized according to the previously described method [13]. Briefly, 1 mmol of citric acid and 3 mmol urea were dissolved in 40 mL H_2O and stirred until a clear solution was formed. The solution was then transferred to a 100 mL Teflon-lined autoclave and the reaction was carried out at 180 ^{0}C for 12 h. The solution was then cooled naturally to room temperature. The product was used for characterization and measurements without additional purification. Based on the synthesis conditions, the estimated material concentration is 9.3 mg/mL.

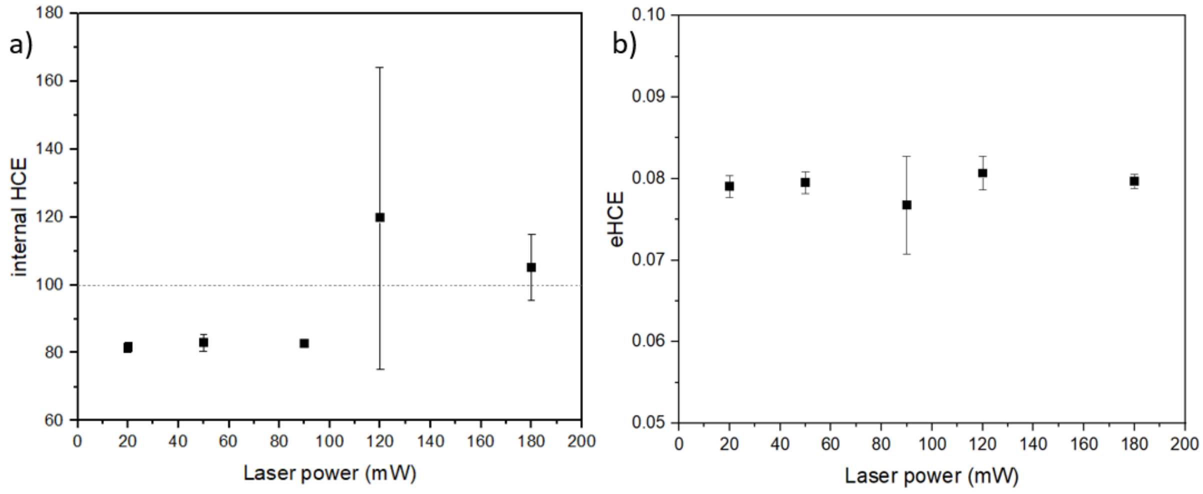

Figure S1. Power dependence of internal HCE and eHCE. Measurements were performed for γ-Fe_2O_3 sample at 806 nm. Error bars are standard deviation from 3 measurements.

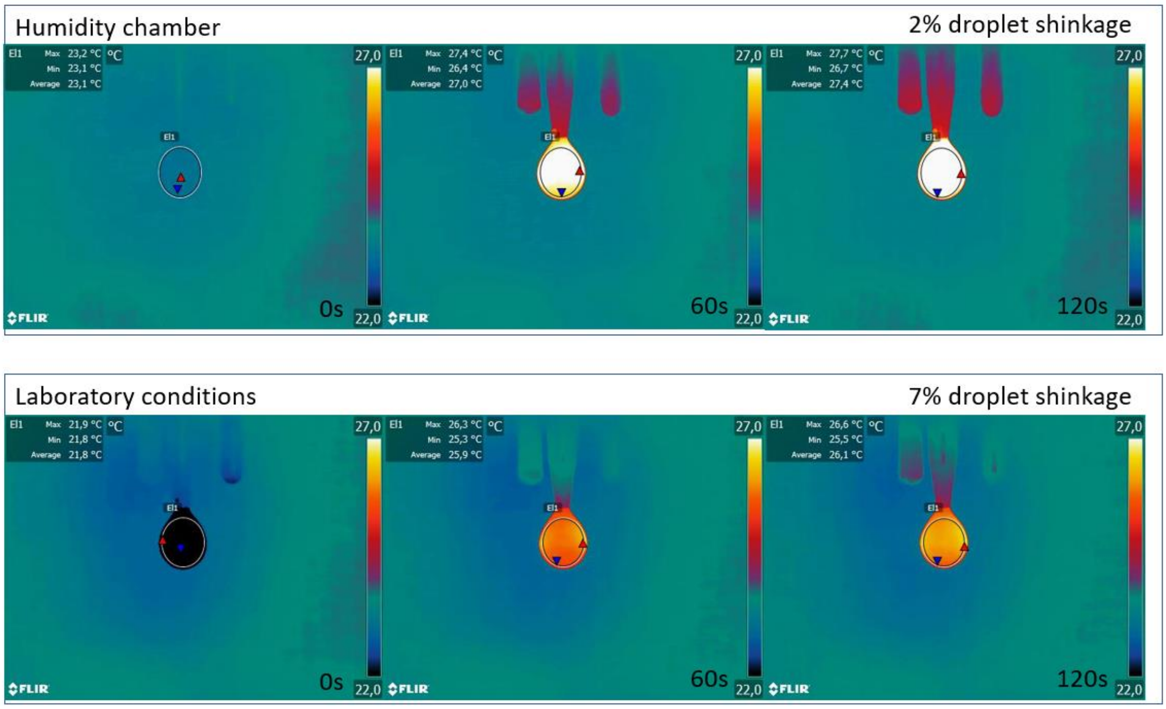

Figure S2. Thermal images of droplets registered at increased (top) and ambient (bottom) humidity conditions at 0 (left), 60 (middle) and 120 (right) seconds of irradiation at 980 nm, 90 mW.

Table S1. Comparison of the possibilities of using different materials in photothermal therapy. Data provided for 794 nm excitation (except for CDs which occurred at 445 nm).

**NH class**	**NH**	**λ with max. of Abs.**	**α (L/(g·cm))**	**iHCE 794 nm (%)**	**eHCE 794 nm (L/(g·cm))**
plasmonic	AuNRs	~780	0.627	~100	0.627
	CuS@citrate	926	4.49	~100	4.49
	CuS@glutathione	1037	3.60	94	3.38
lanthanide-based	$NaNdF_4$@PAA	794	0.0166	74	0.0122
	$NaNdF_4$:50%Dy @PAA	794	0.0106	41	0.0044
	$NaNdF_4$:80%Dy @PAA	794	0.0086	17	0.0014
	$NaNdF_4$:25%Sm @PAA	794	0.0217	60	0.0130
	$NaNdF_4$:50%Sm @PAA	794	0.0067	40	0.0027
	$NaNdF_4$:75%Sm @PAA	794	0.0058	31	0.0017
semiconductors	Black porous silicon	UV	9.92	39	3.84
	Ag-Ag_2S	UV	0.832	96	0.798
	Ag2S@PEG	UV	0.695	81	0.566
	Ag2S@MUA	UV	1.46	83	1.21
	Ag2S@DTDTPA	UV	0.0753	79	0.0598
iron oxide	γ-Fe_2O_3	UV	0.0927	82	0.0763
	γ-Fe_2O_3@Au	UV	0.487	86	0.420
carbon	carbon nanodots	UV	0.338 (445 nm)	~100 (445 nm)	0.338 (445 nm)

Table S2 iHCE of Nd^{3+} doped nanomaterials

**Material, diameter**	**diameter**	**λ [nm]**	**iHCE [%]**	**Ref**
$NaNdF_4$	9-25 nm	800	85-74	[14]
$NaNdF_4$@$NaYF_4$@$NaYF_4$:1%Nd	25 nm	808	72	[15]
$NaNdF_4$@prussian blue	29 nm	808	60.8	[16]
$NaNdF_4$	19 nm	808	8.7	[16]
$NdVO_4$,	2.4 nm	808	72	[17]
$NaErF_4$@$NaYF_4$@$NaNdF_4$ – prussian blue	51.1 nm (without PB), 164 nm	808	50.5	[18]
$NaYF_4$:Yb,Er@$NaYF_4$:Yb @$NaYF_4$:Yb,Nd @$mSiO_2$/IR806@PAH-PEG-FA	41 nm	793	46	[19]
$NdVO_4$/Au	21 nm	808	32.2	[20]

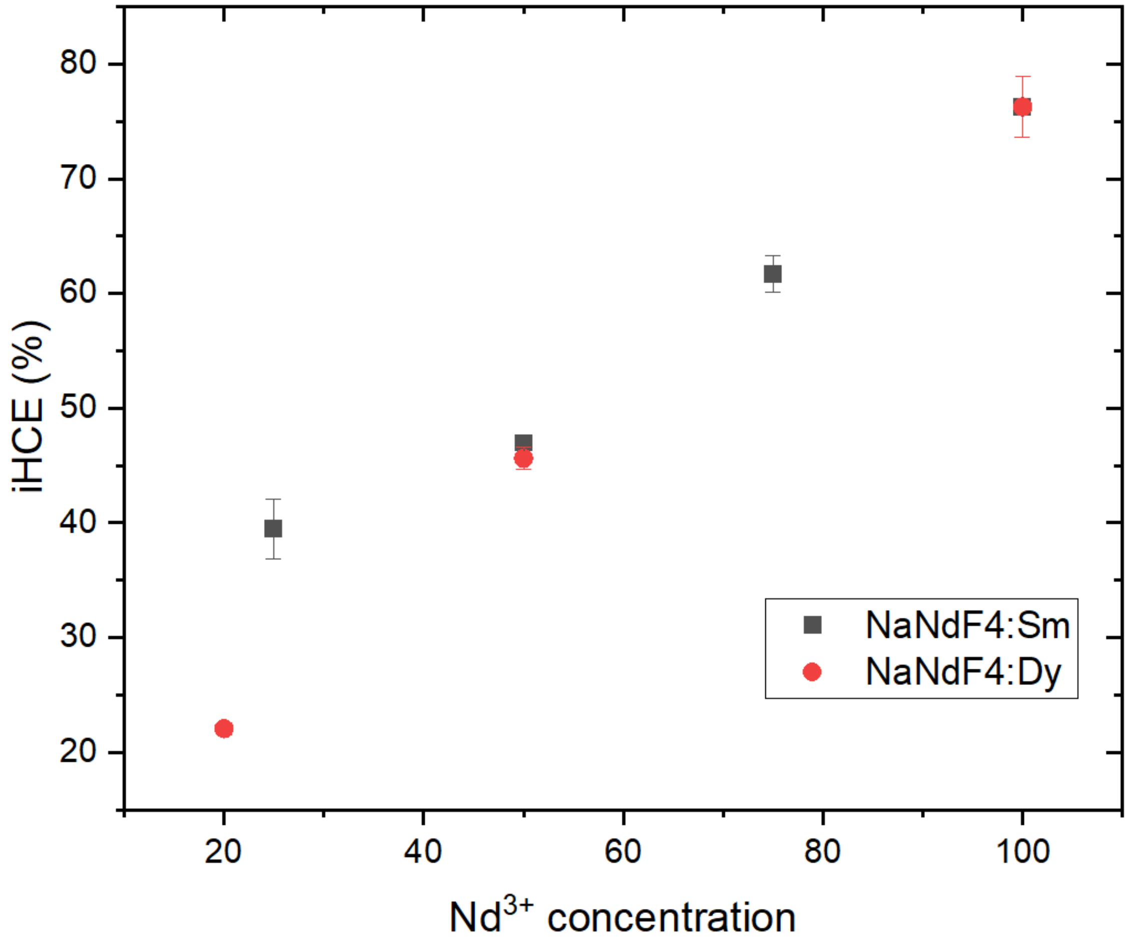

Figure S3. Internal light-to-heat conversion efficiency of $NaNdF_4$ in function of Sm^{3+}/Dy^{3+} dopant concentration.

Table S3. Emission quantum yield under 808 nm CW laser irradiation at distinct laser power densities. The emission quantum yield values were obtained integrating the emission in the range 947.92-1451.33 nm.

Identifier	Power Density ($W.cm^{-2}$)	QY (%)
Ag_2S@PEG	532	$<1\times10^{-5}$
Ag_2S@11MUA	532	0.064±0.006
Ag_2S@DPDPA	532	0.66±0.07

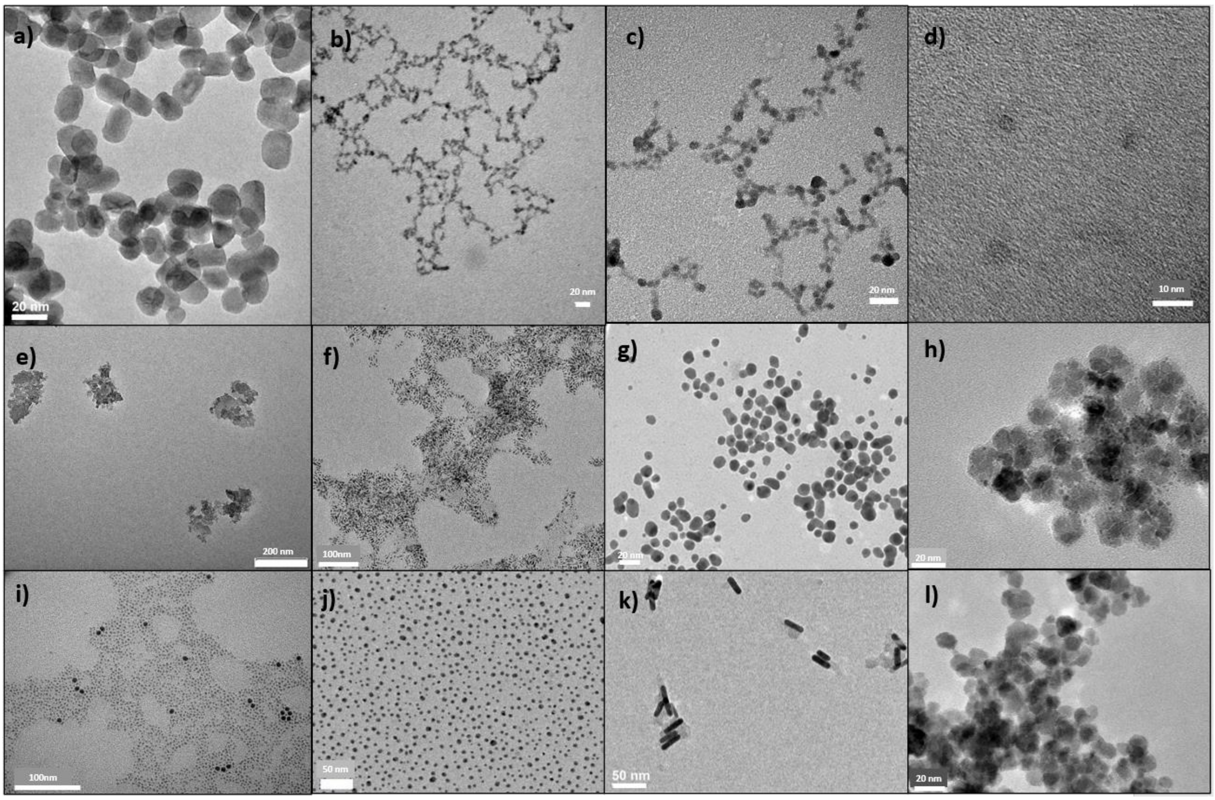

Figure S4 Morphology of the materials compared in this work: a) $NaNdF_4$: 50Sm, b) CuS covered by GSH c) CuS covered by citrate, d) carbon dots, e) black porous silicon, f) Ag_2S@MUA, g) Ag-Ag_2S dimers, h) γ-Fe_2O_3 decorated with gold nanoparticles,

i) Ag_2S@DTDTPA, j) Ag_2S decorated with PEG, k) colloidal gold in the form of nanorods, l) γ-Fe_2O_3

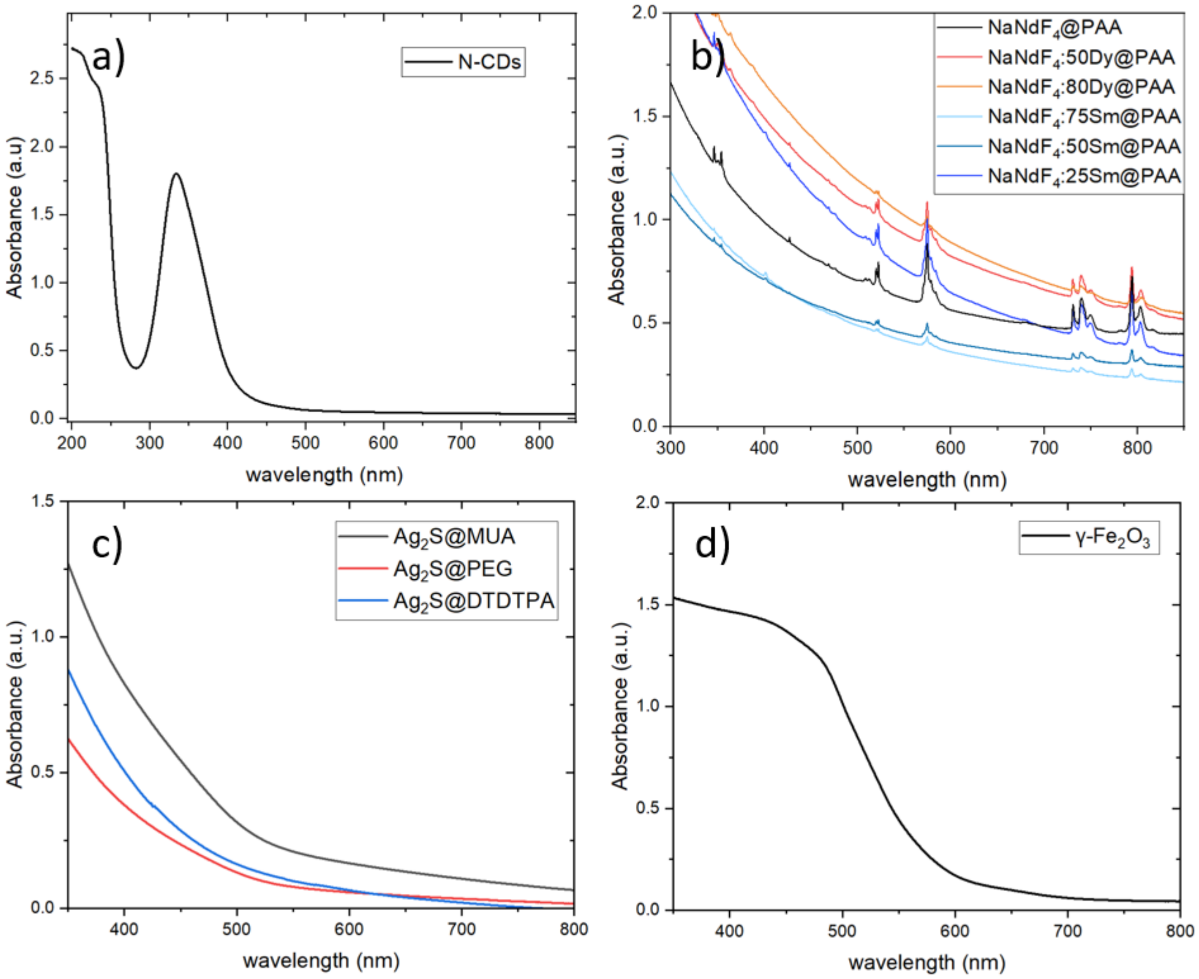

Figure S5. Absorption spectra of a) CQDs, b) $NaNdF_4$ doped with Dy^{3+} and Sm^{3+}, c) Ag_2S, d) γ-Fe_2O_3 . Absorption spectra for γ-Fe_2O_3 decorated with gold nanoparticles, are given in ref of the following article in SI: [11].

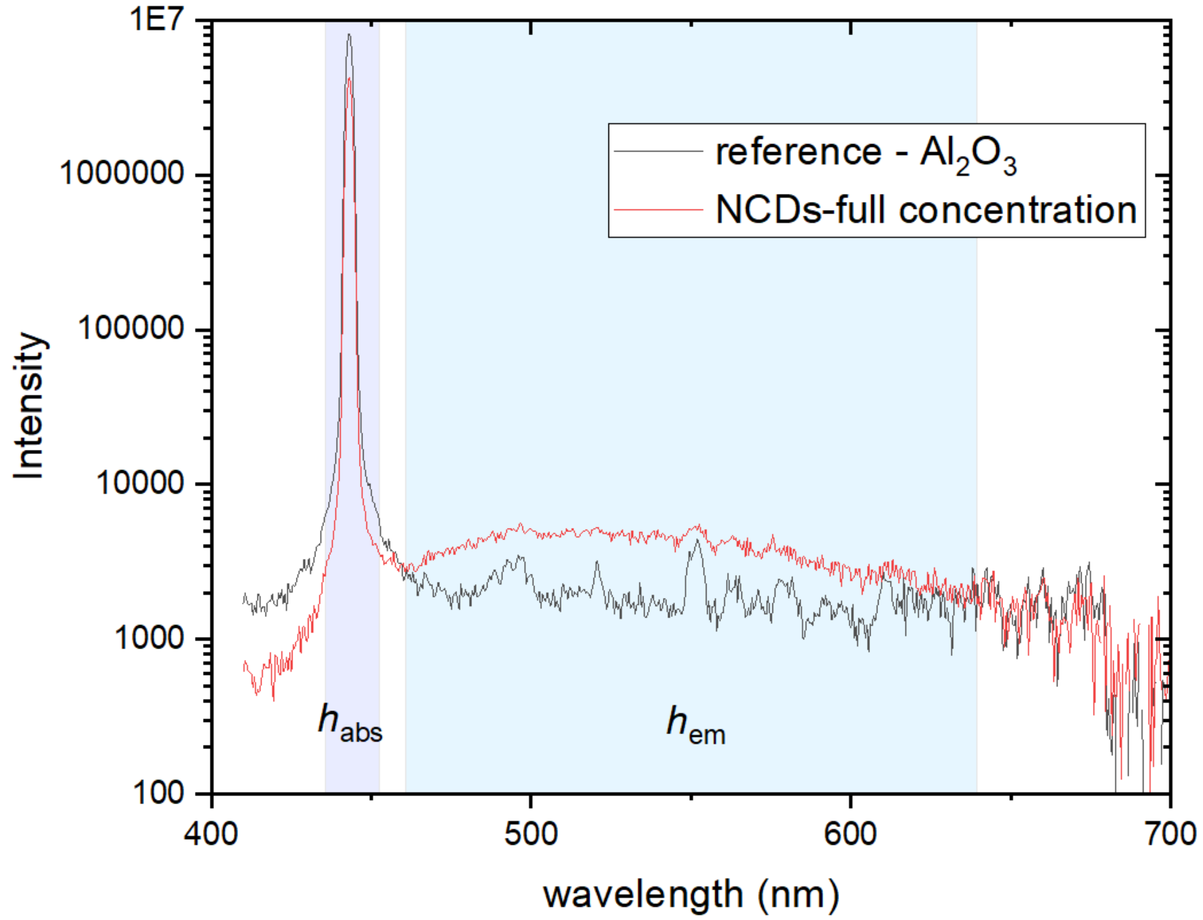

Figure S6. Photoluminescence quantum yield (QY) measurement of CDs. QY was calculated basing on Equation SE1:

$$QY = \frac{h_{em}}{h_{abs}} = \frac{I_{CDs} - I_{ref}}{I_{ref} - I_{CDs}} \quad \text{(SE1)}$$

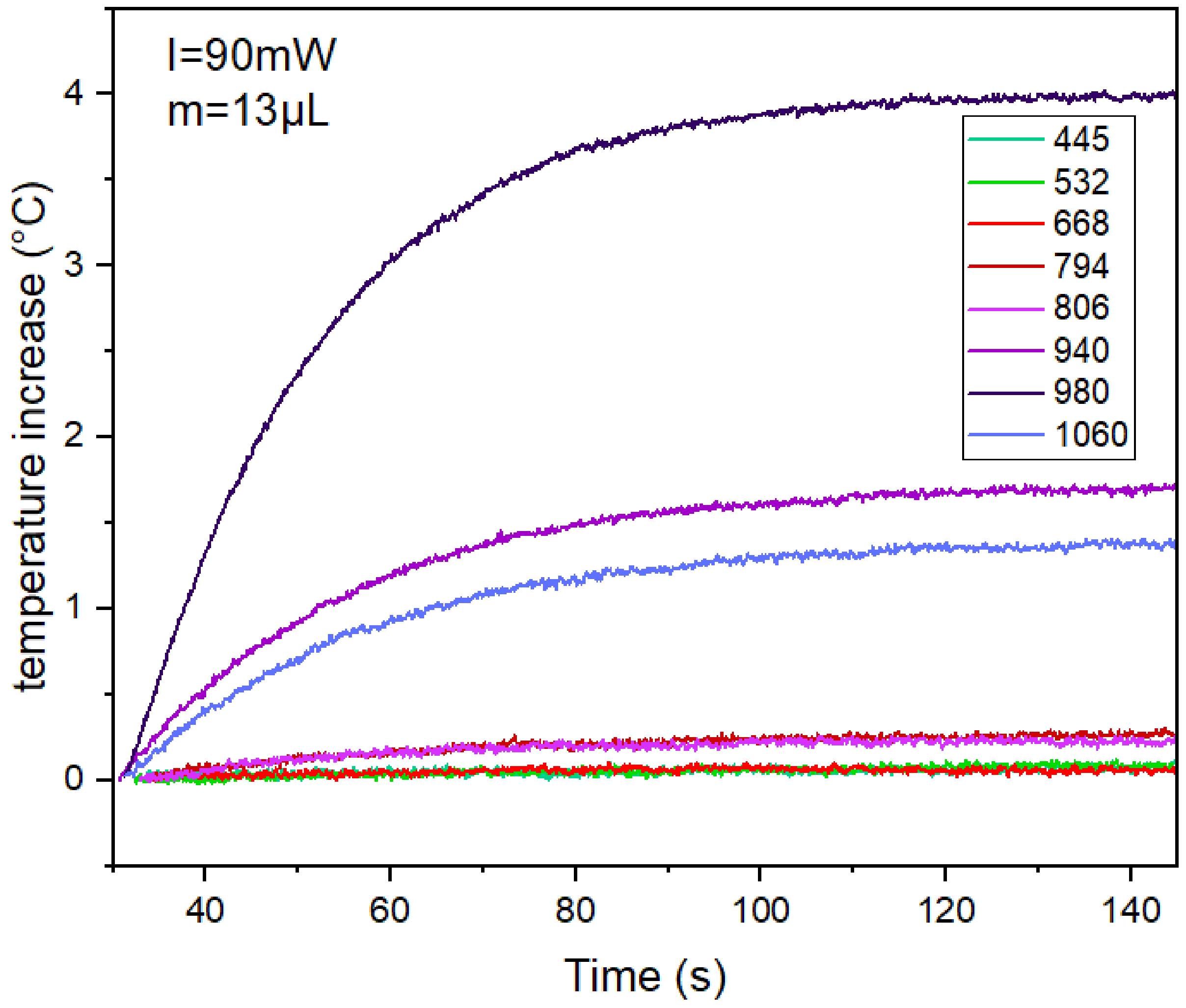

Figure S7. Temperature rise curves of a 13 uL water droplet for different wavelengths measured at constant irradiance power (90 mW).

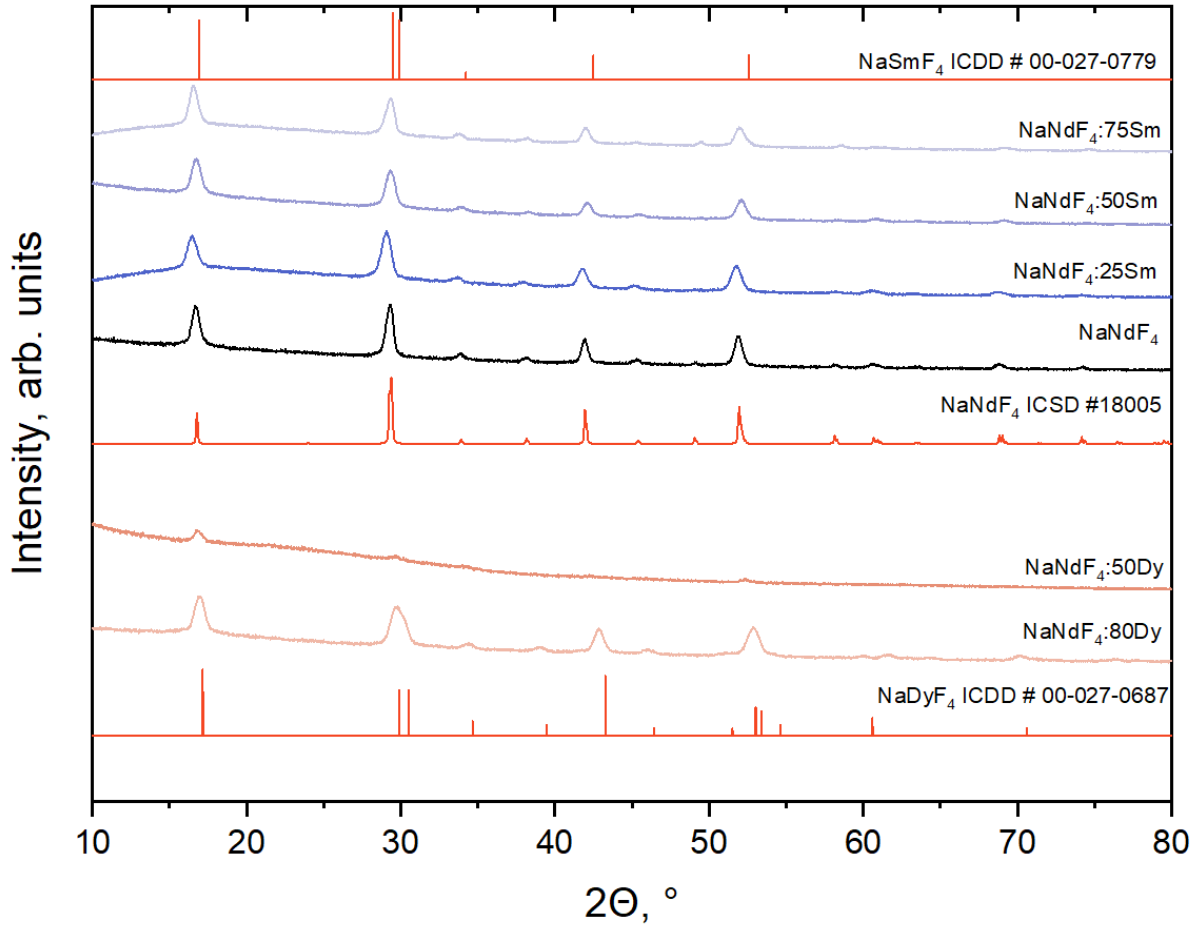

Figure S8 XRD of $NaNdF_4$ samples doped with Dy^{3+} and Sm^{3+}.

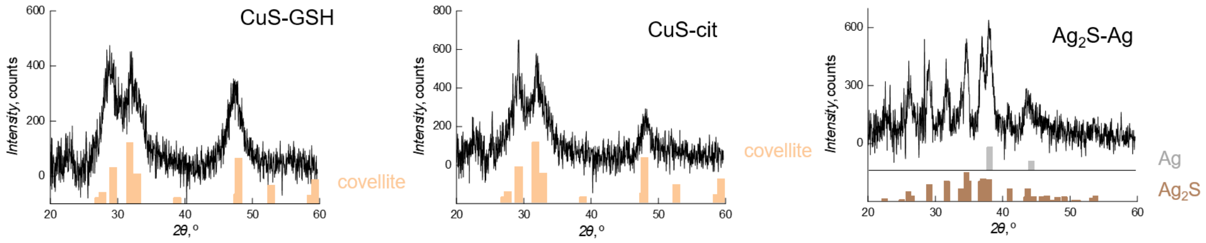

Figure S9 XRD of CuS@GSH, CuS@cit and Ag-Ag_2S samples

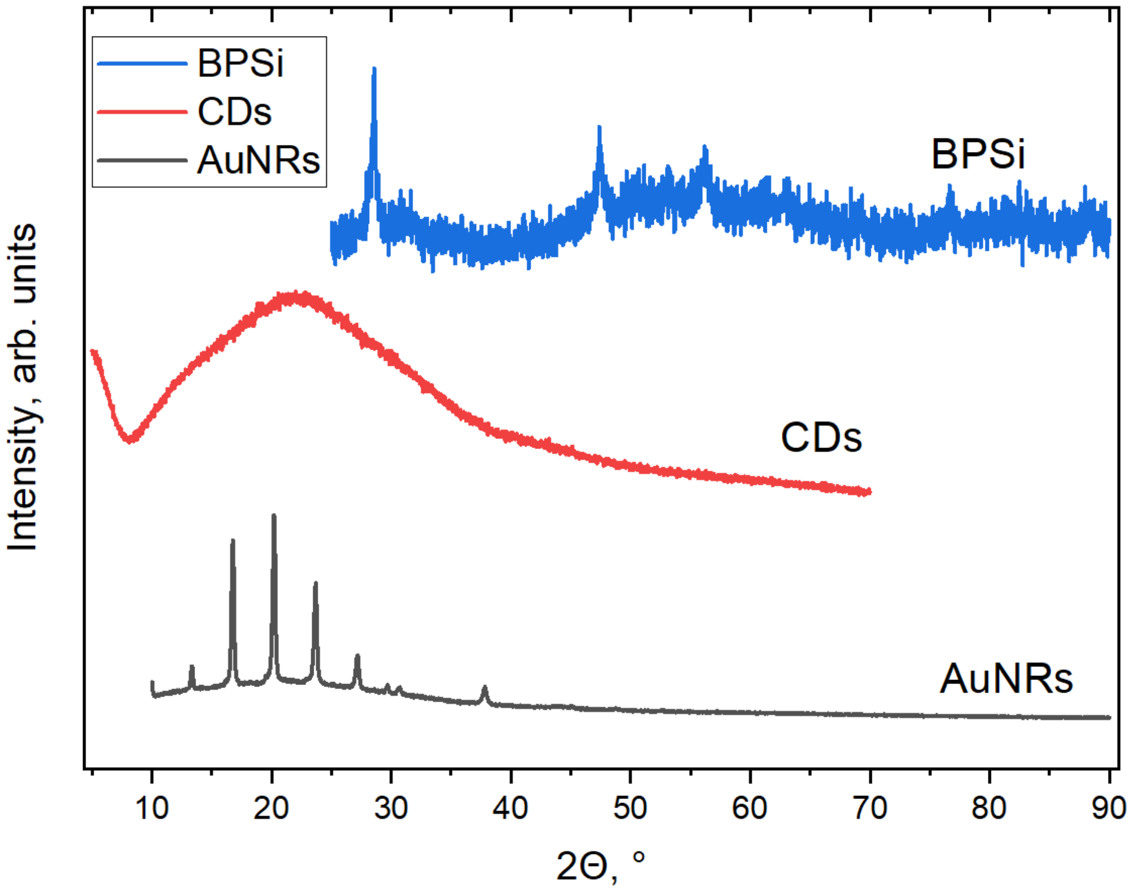

Figure S10 XRD of BPSi, CDs and AuNRs samples

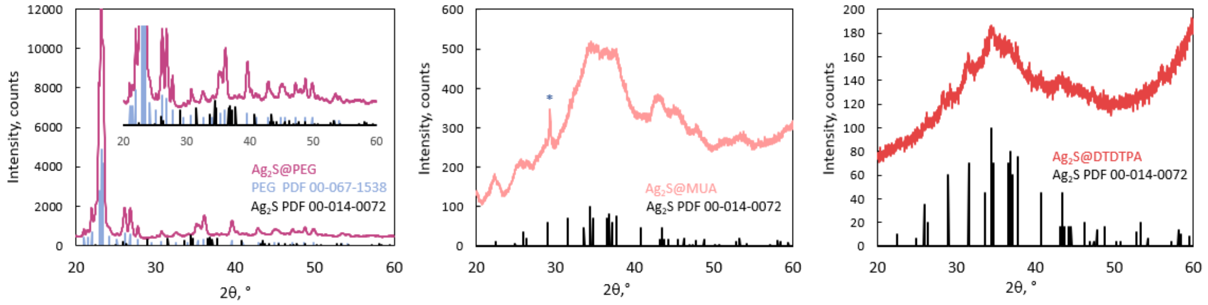

Figure S11 XRD of Ag_2S samples, * is a peak from sample holder.

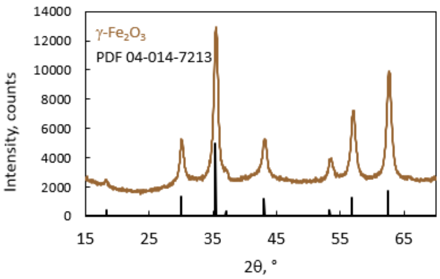

Figure S12 XRD of γ-Fe_2O_3 sample

References:

(1) Arifin, D. R.; Long, C. M.; Gilad, A. A.; Alric, C.; Roux, S.; Tillement, O.; Link, T. W.; Arepally, A.; Bulte, J. W. M. Trimodal Gadolinium-Gold Microcapsules Containing Pancreatic Islet Cells Restore Normoglycemia in Diabetic Mice and Can Be Tracked by Using US, CT, and Positive-Contrast MR Imaging. *Radiology* **2011**, *260* (3), 790–798. https://doi.org/10.1148/radiol.11101608.

(2) Scarabelli, L.; Sánchez-Iglesias, A.; Pérez-Juste, J.; Liz-Marzán, L. M. A "Tips and Tricks" Practical Guide to the Synthesis of Gold Nanorods. *J. Phys. Chem. Lett.* **2015**, *6* (21), 4270–4279. https://doi.org/10.1021/acs.jpclett.5b02123.

(3) Marin, R.; Lifante, J.; Besteiro, L. V.; Wang, Z.; Govorov, A. O.; Rivero, F.; Alfonso, F.; Sanz-Rodríguez, F.; Jaque, D. Plasmonic Copper Sulfide Nanoparticles Enable Dark Contrast in Optical Coherence Tomography. *Adv. Healthc. Mater.* **2020**, *9* (5). https://doi.org/10.1002/adhm.201901627.

(4) Marciniak, L.; Pilch, A.; Arabasz, S.; Jin, D.; Bednarkiewicz, A.; Chen, M.; Sun, Y.; Li, F. Y.; Solé, J. G.; Jaque, D.; Palacio, F.; Carlos, L. D.; Millán, A.; Pedroni, M.; Speghini, A.; Hirata, G. A.; Martin, I. R.; Jaque, D. Heterogeneously Nd $^{3+}$ Doped Single Nanoparticles for NIR-Induced Heat Conversion, Luminescence, and Thermometry. *Nanoscale* **2017**, *9* (24), 8288–8297. https://doi.org/10.1039/C7NR02630G.

(5) Marin, R.; Benayas, A.; García-Carillo, N.; Lifante, J.; Yao, J.; Mendez-Gonzalez, D.; Sanz-Rodríguez, F.; Rubio-Retama, J.; Besteiro, L. V.; Jaque, D. Nanoprobes for Biomedical Imaging with Tunable Near-Infrared Optical Properties Obtained via Green Synthesis. *Adv. Photonics Res.* **2021**, *2100260*, 2100260. https://doi.org/10.1002/adpr.202100260.

(6) Glais, E.; Pellerin, M.; Castaing, V.; Alloyeau, D.; Touati, N.; Viana, B.; Chanéac, C. Luminescence Properties of ZnGa 2 O 4 :Cr 3+ ,Bi 3+ Nanophosphors for Thermometry Applications. *RSC Adv.* **2018**, *8* (73), 41767–41774. https://doi.org/10.1039/c8ra08182d.

(7) Xu, W.; Tamarov, K.; Fan, L.; Granroth, S.; Rantanen, J.; Nissinen, T.; Peräniemi, S.; Uski, O.; Hirvonen, M. R.; Lehto, V. P. Scalable Synthesis of Biodegradable Black Mesoporous Silicon Nanoparticles for Highly Efficient Photothermal Therapy. *ACS Appl. Mater. Interfaces* **2018**, *10* (28), 23529–23538. https://doi.org/10.1021/acsami.8b04557.

(8) Xu, W.; Leskinen, J.; Tick, J.; Happonen, E.; Tarvainen, T.; Lehto, V. P. Black Mesoporous Silicon as a Contrast Agent for LED-Based 3D Photoacoustic Tomography. *ACS Appl. Mater. Interfaces* **2020**, *12* (5), 5456–5461. https://doi.org/10.1021/acsami.9b18844.

(9) Vayssières, L.; Chanéac, C.; Tronc, E.; Jolivet, J. P. Size Tailoring of Magnetite Particles Formed by Aqueous Precipitation: An Example of Thermodynamic Stability of Nanometric Oxide Particles. *J. Colloid Interface Sci.* **1998**, *205* (2), 205–212. https://doi.org/10.1006/jcis.1998.5614.

(10) Jolivet, J. P.; Chanéac, C.; Tronc, E. Iron Oxide Chemistry. From Molecular Clusters to Extended Solid Networks. *Chem. Commun.* **2004**, *4* (5), 477–483. https://doi.org/10.1039/b304532n.

(11) Nicolás-Boluda, A.; Vaquero, J.; Laurent, G.; Renault, G.; Bazzi, R.; Donnadieu, E.; Roux, S.; Fouassier, L.; Gazeau, F. Photothermal Depletion of Cancer-Associated Fibroblasts Normalizes Tumor Stiffness in Desmoplastic Cholangiocarcinoma. *ACS Nano* **2020**, *14* (5), 5738–5753. https://doi.org/10.1021/acsnano.0c00417.

(12) Alric, C.; Miladi, I.; Kryza, D.; Taleb, J.; Lux, F.; Bazzi, R.; Billotey, C.; Janier, M.; Perriat, P.; Roux,

S.; Tillement, O. The Biodistribution of Gold Nanoparticles Designed for Renal Clearance. *Nanoscale* **2013**, *5* (13), 5930–5939. https://doi.org/10.1039/c3nr00012e.

(13) Zhang, X.; Qin, X.; Zhang, W. NaYF4:Yb, Er with N-GQDs Mixture: One-Pot Hydrothermal Synthesis and Its Luminescent Film. *Opt. Mater. (Amst).* **2021**, *114* (February), 110910. https://doi.org/10.1016/j.optmat.2021.110910.

(14) Xu, L.; Li, J.; Lu, K.; Wen, S.; Chen, H.; Shahzad, M. K.; Zhao, E.; Li, H.; Ren, J.; Zhang, J.; Liu, L. Sub-10 Nm NaNdF 4 Nanoparticles as Near-Infrared Photothermal Probes with Self-Temperature Feedback . *ACS Appl. Nano Mater.* **2020**, *3* (3), 2517–2526. https://doi.org/10.1021/acsanm.9b02606.

(15) Marciniak, L.; Pilch, A.; Arabasz, S.; Jin, D.; Bednarkiewicz, A. Heterogeneously Nd3+doped Single Nanoparticles for NIR-Induced Heat Conversion, Luminescence, and Thermometry. *Nanoscale* **2017**, *9* (24), 8288–8297. https://doi.org/10.1039/c7nr02630g.

(16) Yu, Z.; Hu, W.; Zhao, H.; Miao, X.; Guan, Y.; Cai, W.; Zeng, Z.; Fan, Q.; Tan, T. T. Y. Generating New Cross-Relaxation Pathways by Coating Prussian Blue on NaNdF 4 To Fabricate Enhanced Photothermal Agents . *Angew. Chemie* **2019**, *100871*, 8624–8628. https://doi.org/10.1002/ange.201904534.

(17) del Rosal, B.; Pérez-Delgado, A.; Carrasco, E.; Jovanović, D. J.; Dramićanin, M. D.; Dražić, G.; de la Fuente, Á. J.; Sanz-Rodriguez, F.; Jaque, D. Neodymium-Based Stoichiometric Ultrasmall Nanoparticles for Multifunctional Deep-Tissue Photothermal Therapy. *Adv. Opt. Mater.* **2016**, *4* (5), 782–789. https://doi.org/10.1002/adom.201500726.

(18) Wang, X.; Li, H.; Li, F.; Han, X.; Chen, G. Prussian Blue-Coated Lanthanide-Doped Core/Shell/Shell Nanocrystals for NIR-II Image-Guided Photothermal Therapy. *Nanoscale* **2019**, *11* (45), 22079–22088. https://doi.org/10.1039/c9nr07973d.

(19) Lin, S. L.; Chen, Z. R.; Chang, C. A. Nd3+ Sensitized Core-Shell-Shell Nanocomposites Loaded with Ir806 Dye for Photothermal Therapy and up-Conversion Luminescence Imaging by a Single Wavelength Nir Light Irradiation. *Nanotheranostics* **2018**, *2* (3), 243–257. https://doi.org/10.7150/ntno.25901.

(20) Chang, M.; Wang, M.; Shu, M.; Zhao, Y.; Ding, B.; Huang, S.; Hou, Z.; Han, G.; Lin, J. Enhanced Photoconversion Performance of NdVO4/Au Nanocrystals for Photothermal/Photoacoustic Imaging Guided and near Infrared Light-Triggered Anticancer Phototherapy. *Acta Biomater.* **2019**, *99*, 295–306. https://doi.org/10.1016/j.actbio.2019.08.026.
